# Phytoextraction of potentially toxic elements by six tree species growing on hazardous mining sludge

**DOI:** 10.1007/s11356-017-9842-3

**Published:** 2017-08-09

**Authors:** Mirosław Mleczek, Piotr Goliński, Magdalena Krzesłowska, Monika Gąsecka, Zuzanna Magdziak, Paweł Rutkowski, Sylwia Budzyńska, Bogusława Waliszewska, Tomisław Kozubik, Zbigniew Karolewski, Przemysław Niedzielski

**Affiliations:** 1 Department of Chemistry, Poznań University of Life Sciences, Wojska Polskiego 75, 60-625 Poznań, Poland; 2 Faculty of Biology, Laboratory of General Botany, Adam Mickiewicz University in Poznań, Umultowska 89, 61-614 Poznań, Poland; 3 Department of Forest Sites and Ecology, Poznań University of Life Sciences, Wojska Polskiego 71F, 60-625 Poznań, Poland; 4 Institute of Chemical Wood Technology, University of Life Sciences in Poznan, Wojska Polskiego 75, 60-625 Poznań, Poland; 5 Energetyka S.A, M. Skłodowskiej-Curie 58, 59-301 Lubin, Poland; 6 Department of Phytopathology, Poznan University of Life Sciences, Dąbrowskiego 159, 60-594 Poznań, Poland; 7 Faculty of Chemistry, Adam Mickiewicz University in Poznań, Umultowska 89B, 61-614 Poznań, Poland

**Keywords:** Heavy metals, Mining sludge, Phytoextraction, Tree species

## Abstract

The aim of the study was to compare the phytoextraction abilities of six tree species (*Acer platanoides* L., *Acer pseudoplatanus* L., *Betula pendula* Roth, *Quercus robur* L., *Tilia cordata* Miller, *Ulmus laevis* Pall.), cultivated on mining sludge contaminated with arsenic (As), cadmium (Cd), copper (Cu), lead (Pb), thallium (Tl), and zinc (Zn). All six tree species were able to survive on such an unpromising substrate. However, *A. platanoides* and *T. cordata* seedlings grown on the polluted substrate showed significantly lower biomass than control plants (55.5 and 45.6%, respectively). As, Cd, Cu, Pb, and Tl predominantly accumulated in the roots of all the analyzed tree species with the following highest contents: 1616, 268, 2432, 547, and 856 mg kg^−1^, respectively. Zn was predominantly localized in shoots with the highest content of 5801 and 5732 mg kg^−1^ for *U. laevis* and *A. platanoides,* respectively. *A. platanoides* was the most effective in Zn phytoextaction, with a bioconcentration factor (BCF) of 8.99 and a translocation factor (TF) of 1.5. Furthermore, with the exception of *A. pseudoplatanus*, the analyzed tree species showed a BCF > 1 for Tl, with the highest value for *A. platanoides* (1.41). However, the TF for this metal was lower than 1 in all the analyzed tree species. *A. platanoides* showed the highest BCF and a low TF and could, therefore, be a promising species for Tl phytostabilization. In the case of the other analyzed tree species, their potential for effective phytoextraction was markedly lower. Further studies on the use of *A. platanoides* in phytoremediation would be worth conducting.

**Electronic supplementary material:**

The online version of this article (doi:10.1007/s11356-017-9842-3) contains supplementary material, which is available to authorized users.

## Introduction

Landfill sites composed of hazardous wastes represent a significant environmental problem due to their harmful effect on humans, animals, and plants (Mukhacheva et al. 2010; Shobier et al. 2011). In many cases, there are no simple methods for their management and/or limitation of their negative influence on living organisms (Favas et al. 2011; Nanseu-Njiki et al. 2009; Yakout et al. 2016). For this reason, using biological processes such as phytoremediation may offer a chance to change their chemical characteristics and/or decrease toxic element concentrations (Salazar and Pignata 2014; Singh et al. 2016). A number of studies present diverse analysis and comparisons in an attempt to select the most promising plant species to use in the phytoextraction of toxic elements from polluted substrates (Kacálková et al. 2015; Kertulis-Tartar et al. 2006). Phytoremediation as a green technology offers many ecological benefits for the environment, although the long-lasting process (tens or even hundreds of years) still limits its practice (Mleczek et al. 2013). For this reason, the selection of appropriate plant species is of fundamental importance. The most effective plant species are characterized by high efficiency in phytoextraction of elements, translocation to aerial parts, easy adaptation to the presence of pollutants, and relatively high biomass as well as simplified collection procedures for plant material after their growth.

According to literature data, certain tree species possess great potential for practical application in phytoremediation (Escobar and Dussan 2016; Fernández et al. 2016). Typical wood species in particular seem to be preferred due to the high biomass of their aerial parts, low environmental requirements, and high transpiration rate. Among the most satisfactory accumulators of metals are *Populus tremuloides* (Ni, Zn), *Alnus acuminata* subsp. *acuminata* (Cr, Pb), *Populus alba* (Cd, Pb), and *Salix* species (Cd, Zn) (Escobar and Dussan 2016; Houda et al. 2016; Kalubi et al. 2016; Yang et al. 2015).

In extremely contaminated areas, the flora is usually reduced and trees rarely constitute primary colonizing species (Prasad and Freitas 2003). The vegetation consists of plants that possess great potential for phytoextraction or phytostabilization of harmful metals. These plants are characterized by high bioaccumulation factors and/or ability to either tolerate high levels of metals in their organs or exclude potentially toxic elements (Liu et al. 2014). However, the ability to transfer metals from soil/air to plant organs makes some species of trees suitable for the colonization of contaminated areas and suitable for vegetation restoration (Fernández et al. 2016; Sawidis et al. 2011; Ugolini et al. 2013; Yakun et al. 2016). On the other hand, many tree species are considered to be sensitive plants, since they usually grow in clean ecosystems such as forests, often characterized by low concentrations of potentially toxic elements in soil. The high sensitivity of selected tree or bush species has been the reason for the rapid development of studies on plant species such as poplar, willow, mulberry, eucalyptus, legumes, or different grasses (Luo et al. 2016; Kalubi et al. 2016; Lingyun et al. 2015; Singh et al. 2016). In general, fast-growing plant species (*Paulownia elongata*, *Populus nigra*, or *Salix* × *matsudana* × *alba*) are characterized by rapid but limited growth, while typical forest tree species, due to their much longer growth period and high biomass, may be ideal for phytoremediation purposes. Trees have been suggested as low-cost, sustainable, and eco-friendly solutions for the remediation of heavy metal-contaminated lands (Dickinson 2000), especially when other treatments are uneconomical or there is no time pressure on the reuse of the land (Riddell-Black 1994).

Trees possess many valuable qualities with respect to the phytoextraction process along with widespread root systems that are able to penetrate substrate and large biomass production (Pulford et al. 2001). All these beneficial characteristics for phytoremediation technology inspired us to test the phytoextraction efficiency of six tree species growing on highly polluted mining sludge by assessing their phytoextraction ability in cultures similar to open air conditions. For this purpose, we selected the following tree species: *Acer platanoides* L., *Acer pseudoplatanus* L., *Ulmus laevis* Pall., *Quercus robur* L., *Betula pendula* Roth, and *Tilia cordata* Miller. The aim of this study was to evaluate the ability of these six tree species to adapt to new growth conditions, their biomass production, and their potential for effective phytoextraction of six potentially toxic elements. This manuscript is a development of our previous paper (Mleczek et al. 2016), where we studied the same tree species growing under controlled conditions in a similar mining sludge. However, in this study, we focused on the analysis of selected element contents in studied plant organs and on how these trees respond during phytoextraction in an open air experiment.

## Materials and methods

### Experimental materials

Experimental plant materials were 2-year-old leafless seedlings of the following tree species: *A. platanoides* L. (Norway maple), *A. pseudoplatanus* L. (sycamore), *B. pendula* Roth (silver birch), *Q. robur* L. (English oak), *T. cordata* Miller (linden), and *U. laevis* Pall. (white elm)—typical tree species for European forests. All seedlings were obtained from the forest nursery of the Pniewy Forest Division (52° 29′ 4″ N, 16° 15′ 28″ E). The soil where the plants were grown had the following characteristics: pH 4.6–7.4, P content 2.60–4.63 g kg^−1^, K 5.57–6.62 g kg^−1^, total N 0.19–0.29%, total C 2.2–3.2%, and organic matter 3.8–5.6%, depending on the tree species and the specific requirements (e.g., pH of soil) indispensable for their proper development (Mleczek et al. 2016).

### Experiment design

**Fig. 1:**
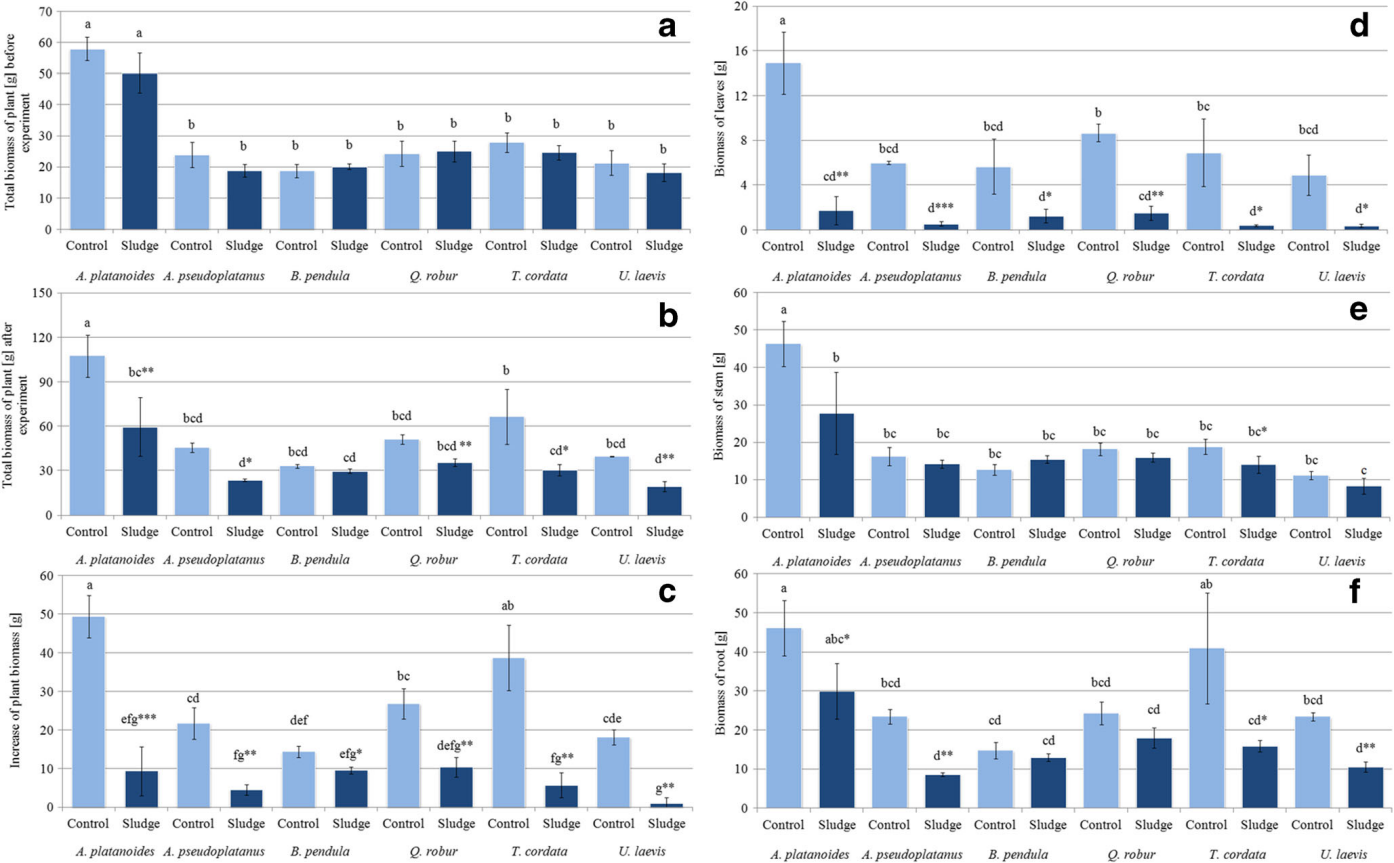
Total plant and organ biomass (g) of tree species grown in soil (*Control*) and mining sludge (*Sludge*). Mean values (*n* = 3); *identical superscripts* (*a*, *b*, *c*…) denote no significant (*p* < 0.05) difference between mean biomass of all observations (separately for each organ of all control and treated tree species), according to Tukey’s HSD test (ANOVA); statistically significant differences between mean biomass of particular control and tree species treated with the *t* test are indicated by an *asterisk*

Tree seedlings were transported to the laboratory in pots filled with soil and were washed with distilled water to remove substrate particles adsorbed on the root system. The plants were then drained, dried with paper towels, and weighed to determine their biomass at the beginning of the experiment (Fig. 1a). All the tree seedlings were then planted on 21 March 2016 in white cylindrical pots (21 × 21 cm, diameter × height) containing 3.9 kg of mining sludge (one plant in one pot and eight plants per tree species) whose characteristics are presented in Table 1.

**Table 1:** Content of elements (mg kg^−1^ DW) in soil and mining sludge compared to data indicating the highest concentration of elements in European soils

Element/form	Soil	Mining sludge	The highest value^a^	Element	Soil	Mining sludge	The highest value^a^
Ag	bDL	24	3.15	Mn	19	633	0.778^b^
Al	421	2005	26.7	Mo	0.23	122	21.3
As_total_	0.24	18,022	282	Na	299	1505	4.45^b^
As(III)	bDL	1766	nad	Nb	bDL	8.57	134
As(V)	bDL	16,220	nad	Nd	4.23	2.63	132
As_org_	bDL	36	nad	Ni	0.75	359	2690
Au	bDL	0.03	nad	Os	bDL	97.7	nad
B	0.79	432	nad	Pb	4.99	3865	970
Ba	7	12	1870	Pd	0.01	0.13	nad
Be	bDL	0.55	18.4	Pr	0.17	20.2	31.6
Bi	0.11	291	9.57	Pt	0.06	369	nad
Ca	3702	95,063	47.7^b^	Rb	bDL	8.82	390
Cd	0.21	1030	14.1	Re	0.01	0.23	nad
Ce	2.99	2.61	267	Rh	bDL	0.28	nad
Co	0.51	74.5	249	Ru	0.01	0.31	nad
Cr	1.32	1260	6230	Sb	0.13	300	31.1
Cs	bDL	773	69.1	Sc	0.13	2.94	54.1
Cu	1.33	4511	256	Se	0.02	0.48	nad
Dy	bDL	bDL	44.9	Si	1.1^c^	4226	96.7^b^
Er	0.98	12.8	26.0	Sm	bDL	0.18	30
Eu	0.03	1.08	6.99	Sn	bDL	1452	106
Fe	1188	31,748	22.3^b^	Sr	2.45	63.2	3120
Ga	bDL	bDL	34.3	Ta	bDL	220	6.78
Gd	0.05	4.97	36.0	Tb	bDL	bDL	7.01
Ge	0.01	0.22	nad	Te	0.19	33.2	0.93
Hf	bDL	bDL	21.2	Th	bDL	48.7	75.9
Hg	0.08	100	1.35	Ti	bDL	143	5.45
Ho	0.02	0.26	9.16	Tl	0.03	669	24.0
In	0.01	17.1	0.41	Tm	0.11	3.19	4.03
Ir	0.13	16.4	nad	U	bDL	bDL	53.2
K	5978	954	6.13^b^	V	0.08	31.08	537
La	0.12	5.59	143	Y	bDL	bDL	267
Li	0.29	2.74	nad	Yb	0.11	2.318	25.0
Lu	0.02	0.43	3.21	Zn	18	1565	2900
Mg	245	5644	24.6^b^	Zr	bDL	40.6	1060

*bDL* below detection limit, *nad* not available data

^a^According to the Geochemical Atlas of Europe (Salminen et al. 2005)

^b^Concentration in % of oxide forms

^c^value in %

The soil used in the experiment had geochemical characteristics similar to those of Polish soils, with respect to element content (Kabata-Pendias and Pendias 1999; Siebielec et al. 2012), whereas the mining sludge was considerably enriched with many elements. A particularly high content of As, Ba, Cd, Cr, Cs, Cu, Hg, Os, Pb, Pt, Sb, Sn, Ta, Tl, and Zn was found. Additionally, a low concentration of methylated arsenic forms in relation to inorganic forms was determined, as described by Francesconi and Kuehnelt (2002).

The control samples comprised the same tree species cultivated in 5.9 kg of soil per pot (higher mass but the same volume because of the lower density of the soil in comparison with the sludge). The control soil was collected from a location free from the direct influence of industrial pollutants (52° 33′ 4″ N, 17° 06′ 20″ E).

Both substrates (soil and mining sludge) were homogenized before the experiment using a POLYMIX PX MFC 90 D/H homogenizer (Kinematica, Inc., NY, USA). In spite of the difference in density of the two substrates, the amount of distilled water added to pots was the same (1.1 L), and water was kept at the same level for the whole duration of the experiment.

All pots were placed on a flat surface (150 m^2^) covered by 8-mm gauge insulating black foil. The tested tree species were arranged so that the distance between every two pots was 1 m. This arrangement (12 × 12 pots) was selected to ensure the same degree of solar irradiation for all plants, easy access for watering, and elimination of the mutual influence of plants (shadowing).

The experiment was conducted in an open area with mean values of temperature of 12.5 °C (ranging between 8.8 and 25.5 °C), air humidity at 68%, and atmospheric pressure of 1003 hPa. Throughout the experiment, plants were watered using deionized water (Milli-Q Academic System (non-TOC), Merck Millipore, Darmstadt, Germany) in amounts appropriate to allow pots to be permanently filled up to 50% of their total volume, independently of tree species. According to data presented by the Provincial Environmental Protection Inspectorate in Poznań, the mean concentration of SO_2_, NO_2_, NO, O_3_, and CO_2_ during the experiment was as follows (μg m^−3^): 4.3, 24.5, 9.5, 61, and 279. The 90-day experiment finished on 18 June 2016.

### Selection of analyzed elements

To determine the 67 elements, an optical emission spectrometer with excitation by inductively coupled plasma Agilent 5100 ICP-OES (Agilent, USA) was used. Of all the 67 elements, 47 of them (Al, As, B, Ba, Bi, Ca, Cd, Ce, Co, Cr, Cu, Er, Eu, Fe, Gd, Ge, Hg, Ho, In, Ir, K, La, Li, Lu, Mg, Mn, Mo, Na, Nd, Ni, Pb, Pd, Pr, Pt, Re, Ru, Sb, Sc, Se, Si, Sr, Te, Tl, Tm, V, Yb, and Zn) were present in soil and mining sludge above the limit of detection of the optical emission spectrometer Agilent 5100 ICP-OES in all analyzed samples, whereas the following other elements in mining sludge were not detected: Ag, Au, Be, Cs, Dy, Ga, Hf, Nb, Os, Rb, Rh, Sm, Sn, Ta, Tb, Th, Ti, U, Y, and Zr in soil and Dy, Ga, Hf, Tb, U, and Y. Only 6 elements (As, Cd, Cu, Pb, Tl, and Zn) out of 67 were chosen for this study because of their potential toxicity, high concentration in the mining sludge, and wide diversity of concentration in the tree organs. Additional information about the concentration of elements useful for plant growth and development (Ca, K, Mg, Na) and also some other elements (Al, B, Ba, Bi, Cr, Fe, Mn, Ni, Pt, Rb, Sr, and V) present in roots, stem, and leaves are reported as Supplementary data (Tables S1–S3, respectively, and Figs. S1 and S2).

### Sample preparation

At the end of the experiment, the plants were washed using ultrapure water (Milli-Q, Millipore, Saint Louis, USA) in order to remove the remaining soil/mining sludge particles from the roots and the adsorbed element ions from the leaf surfaces. To remove excess water, plants were gently dried on filter paper. Whole plants were then weighed and divided into leaves, stems, and roots to estimate the biomass of particular organs. Each plant organ was dried using an electric oven (SLW 53 STD, Pol-Eko, Wodzisław Śląski, Poland). Dry materials were cut in a Cutting Mill SM 200 (Retsch GmbH, Haan, Germany) for 6 min until a powder was obtained. Three representative samples for each organ of all tree species able to grow were weighed (0.3000 ± 0.0001 g), transferred to 55-mL vials, and finally mixed with 8 mL of 65% HNO_3_ (Sigma-Aldrich, St. Louis, MO, USA). Digestion was performed using a CEM Mars 5 Xpress (*CEM*, Matthews, NC, USA) microwave mineralization system. The solutions obtained after digestion were filtered using paper filters (Qualitative Filter Papers Whatman, Grade 595: 4–7 μm) and collected in polypropylene flasks. Solutions were then diluted with deionized water to a final volume of 50 mL.

### Instruments

A simultaneous axial and radial view of the plasma was allowed by the synchronous vertical dual view (SVDV). Common conditions were applied for multielemental determination: radio frequency (RF) power 1.2 kW, nebulizer gas flow 0.7 L min^−1^, auxiliary gas flow 1.0 L min^−1^, plasma gas flow 12.0 L min^−1^, viewing height for radial plasma observation 8 mm, detector charge coupled device (CCD) temperature −40 °C, and signal accusation time 5 s for three replicates.

### Analytical method validation

The detection limits have been determined at the level of 0.0*x* mg kg^−1^ dry weight (DW) or better for all elements determined (as 3-sigma criteria, Table S4). Uncertainty for total analytical procedure (including sample preparation) was at the level of 20%. The analytical efficiency was checked using reference materials CRM S-1—loess soil; CRM NCSDC (73349)—bush branches and leaves; CRM 2709—soil; CRM 405—estuarine sediments; CRM 667—estuarine sediments, and the recovery (80–120%) was acceptable for most of the elements determined (Table S5). For uncertified elements, the recovery was defined with the standard addition method.

### Calculation of phytoextraction efficiency

The efficiency of the analyzed tree species for phytoextraction was estimated based on the bioconcentration factor (BCF) and the translocation factor (TF). BCF, which indicates the efficiency of a plant species in accumulating a metal into its tissues from the surrounding environment, was calculated as the ratio of the concentration of the target metal in the plant to the concentration of the target metal in soil/substrate. TF, which indicates the efficiency of the plant in the translocation of the accumulated metal from its roots to shoots, was calculated as the ratio of the concentration of metal in the shoot to the metal concentration in roots (Ali et al. 2013).

### Statistical analysis

All results were analyzed using STATISTICA 12.0 software (StatSoft, USA). One-way analysis of variance (ANOVA) followed by the post hoc Tukey HSD test was applied to show the differences between biomass or content of selected elements in roots, stems, and leaves of six tree species jointly growing on the mining sludge and soil (control) (Ott 1984). Additionally, to compare the biomass or element contents in the organs of particular tree species growing on mining sludge and in the control, a Student’s *t* test was used (Chambers 1992).

## Results

### Biomass and morphological observations of tree species

At the beginning of the experiment, seedlings of the same species planted in mining sludge and in the control soil were characterized by similar average biomass production. There were no significant differences among the tested tree species with the exception of *A. platanoides* seedlings, characterized by significantly higher biomass than the other plant species (Fig. 1a).

After 90 days of growing on control soil or on sludge substrate, the total biomass of *A. platanoides*, *A. pseudoplatanus*, *B. pendula*, *Q. robur*, *T. cordata*, and *U. laevis* seedlings growing on mining sludge was significantly lower than in the control (55.5, 51.2, 69.3, 45.6, and 48.7% of the biomass of the control plants, respectively) (Fig. 1b). For *B. pendula*, the biomass of the treated plants was not significantly lower than the control seedlings (89.9% of control plant biomass) (Fig. 1b).

The increase of the biomass of the control tree seedlings after the experiment ranged from 43.2% for *B. pendula* to 58.1% for *T. cordata*, while for tree seedlings growing on mining sludge, it ranged from 5.1% for *U. laevis* to 32.1% for *B. pendula*, if compared with their total biomass before the experiment (Fig. 1c).

*A. platanoides* growing in the control soil showed the highest mean biomass of stems (46.3 ± 6.0 g) and leaves (14.9 ± 2.8 g) (Fig. 1d–f), which was probably the effect of the significantly higher biomass of the seedlings of this particular tree species before the experiment (Fig. 1a). It is worth noting that the same relationships for *A. platanoides* grown on mining sludge were not observed. The biomass of the leaves of sludge-grown *A. platanoides*, *A. pseudoplatanus*, *Q. robur*, and *T. cordata* was significantly lower compared to that of the control seedlings (11.7, 9.3, 17.3, and 5.9% of control, respectively).

The biomass of the stems of sludge-treated *A. platanoides* was only 60% of the biomass of the stems of control plants, being the only significant difference for stems (Fig. 1e). Interestingly, the biomass of the stems of *B. pendula* growing on mining sludge was comparable or even higher than the stems of the control seedlings, which might suggest a weak stimulation of the growth of this organ.

*A. platanoides* and *T. cordata* species growing in the control soil were characterized by the highest root biomass (46.1 ± 7.0 and 40.9 ± 14.2 g, respectively) (Fig. 1f), while the biomass of the roots of sludge-grown seedlings of these tree species was significantly lower than the control root biomass (65.0 and 38.9%, respectively). The biomass of treated *A. pseudoplanatnus*, *Q. robur*, and *U. laevis* roots was also significantly lower than that of the control plants (36.9, 74, and 44.9% of the control, respectively). The obtained results related to biomass production (not only of whole tree seedlings but also of their particular organs) indicated the negative influence of mining sludge, especially on leaf amount and on root development.

It is worth underlining that the first visible symptoms of the negative influence of mining sludge on plant development appeared 22 days after the start of the experiment. After this time, the differences between the plants have become more and more evident, especially in leaf development. The toxic effect of the highly polluted mining sludge became visually recognizable after 75–77 days. In particular, the main negative visible symptom was the appearance of chlorotic areas on the leaf blade (Fig. S3a, b). This symptom was observed in the majority of the planted tree species of *A. platanoides*, *B. pendula*, and *U. laevis*.

### Phytoextraction of selected toxic elements

**Fig. 2:**
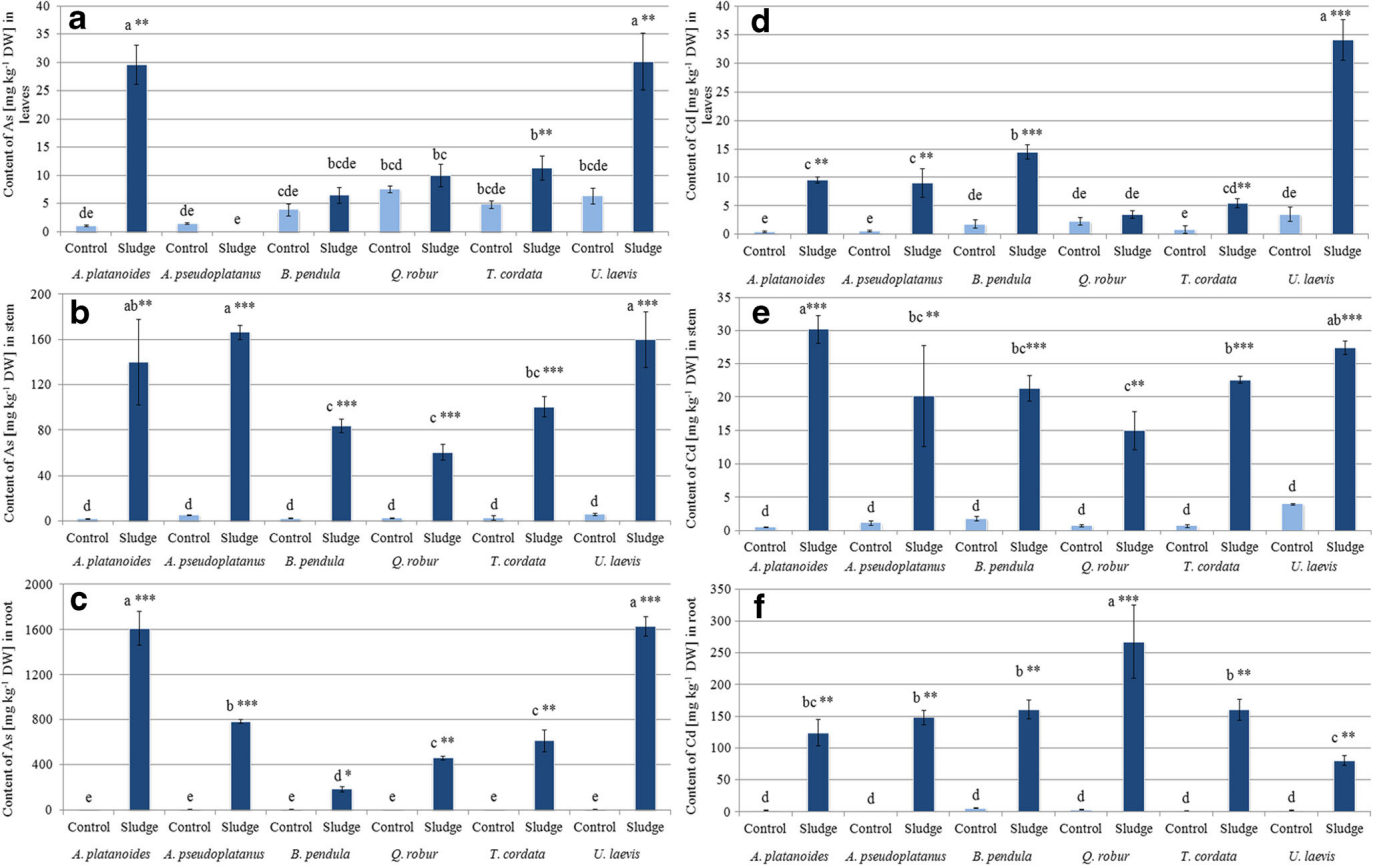
Content of arsenic and cadmium (mg kg^−1^ DW) in root, stem, and leaves of tree species growing in soil (*Control*) and mining sludge (*Sludge*). Mean values (*n* = 3); *identical superscripts* (*a*, *b*, *c*…) denote no significant (*p* < 0.05) difference between mean content of As and Cd of all observations (separately for each organ of all control and treated tree species), according to Tukey’s HSD test (ANOVA); statistically significant differences between mean content of As and Cd of particular control and tree species treated with the *t* test are indicated by an *asterisk*

Generally, the content of As, Cd, Cu, Pb, Tl, and Zn varied both among tree species and/or tree organs. For all the mentioned elements, a significantly higher content was observed in plants growing on mining sludge (Fig. 2).

The highest As concentrations were recorded in *A. platanoides* and *U. laevis*, growing on mining sludge*.* The content of As in their leaves was at least twofold higher than in the other analyzed tree species (Fig. 2a–c).

The As content in *A. platanoides* and *U. laevis* was 1616 ± 152 and 1631 ± 88 mg kg^−1^ in the roots, 29.6 ± 3.5 and 30.2 ± 5.0 mg kg^−1^ in the stems, and 29.6 ± 3.5 and 30.2 ± 5.0 mg kg^−1^ in the leaves, respectively. The As content in roots, stems, and leaves of the other tested tree species was significantly lower (Fig. 2a), with the exception of *A. pseudoplatanus* stems where its content was the highest among the stems of all the tested tree species (167 ± 7 mg kg^−1^). The As content in the roots and stems of all treated sludge-grown seedlings was significantly higher compared to the control plants. In the case of leaves, the same observation was true only for *A. platanoides* and *U. laevis*. *A. platanoides* was able to accumulate the highest amount of As in its whole biomass (mean 52.4 mg per plant). It should be emphasized that 3.95 mg per plant (only 8% of total As plant content) was accumulated in its aboveground organs. Two other effective accumulating tree species were *T. cordata* and *U. laevis*, with an average As content of 11.2 and 18.5 mg per plant, but with a similarly low amount of this metalloid in their shoots (13 and 7%, respectively).

Cadmium concentration varied either among the tested tree species or their organs (Fig. 2d–f). *Q. robur* displayed the highest concentrations for this metal*.* In fact, the highest content of Cd (268 ± 57 mg kg^−1^) was detected in *Q. robur* roots, 40% higher than *B. pendula* and *T. cordata* and 69.8% higher than *U. laevis*. At the same time, *Q. robur* showed the lowest levels of Cd in stems and leaves (15.0 ± 2.8 and 3.6 ± 0.6 mg kg^−1^, respectively). The highest content of Cd in stems was found in *A. platanoides* (30.2 ± 2.1 mg kg^−1^) and *U. laevis* (27.4 ± 1.0 mg kg^−1^). Additionally, *U. laevis* contained the highest amount of Cd (34.2 ± 3.6 mg kg^−1^) in its leaves.

Similarly to As, seedlings of all tested tree species grown on sludge contained significantly higher amounts of Cd than the control plants, especially in their roots and stems (Fig. 2d–f). Only *Q. robur*, the tree species with the highest content of Cd in the whole biomass (5.1 mg per plant), showed no significant differences in Cd accumulation in leaves between the control plants and plants grown on sludge.

**Fig. 3:**
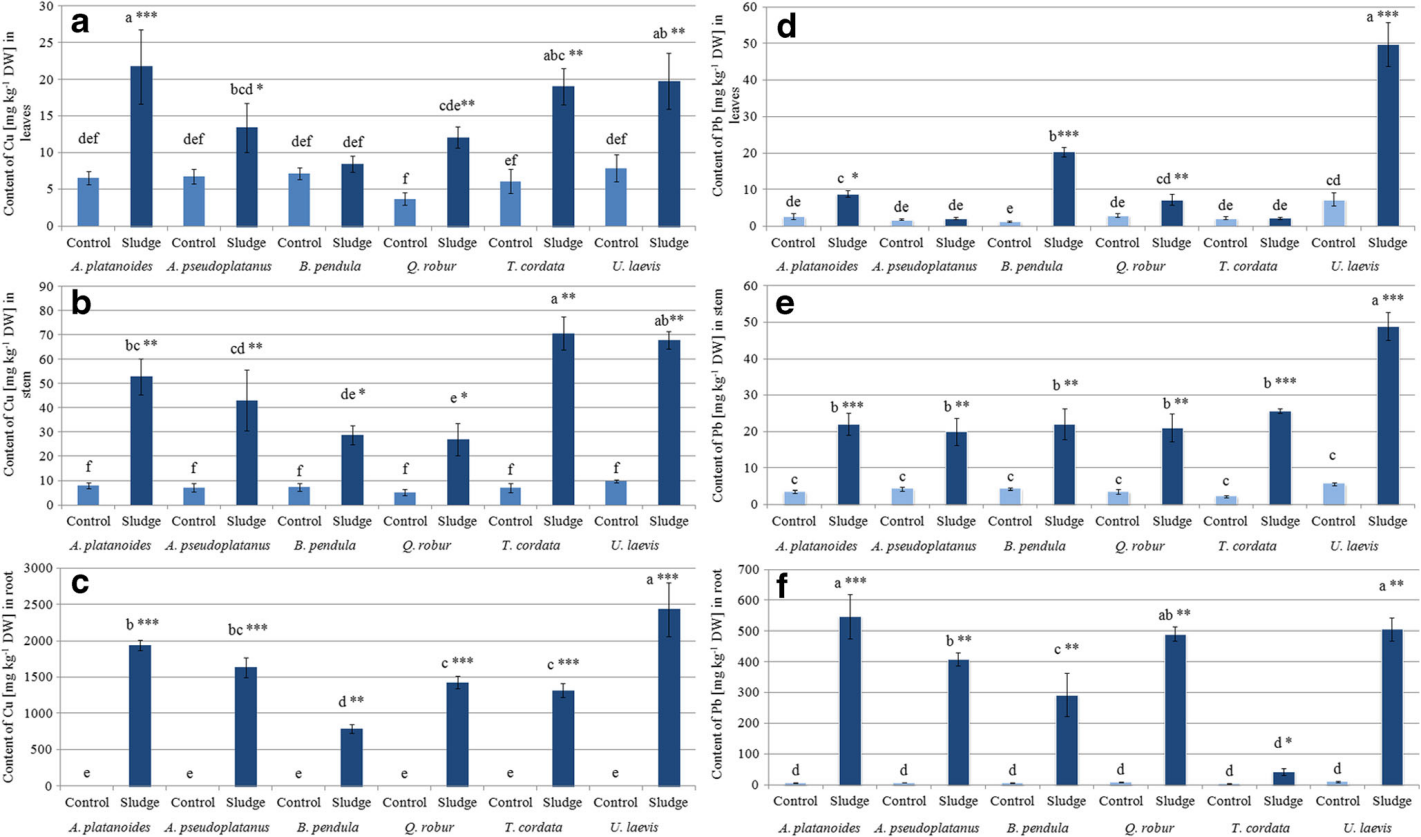
Content of copper and lead (mg kg^−1^ DW) in root, stem, and leaves of tree species growing in soil (*Control*) and mining sludge (*Sludge*). Mean values (*n* = 3); *identical superscripts* (*a*, *b*, *c*…) denote no significant (*p* < 0.05) difference between mean content of Cu and Pb of all observations (separately for each organ of all control and treated tree species), according to Tukey’s HSD test (ANOVA); statistically significant differences between mean content of Cu and Pb of particular control and tree species treated with the *t* test are indicated by an *asterisk*

Copper concentrations in plant biomass are reported in Fig. 3a–c. The highest Cu concentrations were observed in the organs of *A. platanoides*, *Q. robur*, *T. cordata*, and *U. laevis* (Fig. 3a–c). In particular, the highest content of Cu was observed in *U. laevis* and *A. platanoides* roots (2432 ± 370 and 1941 ± 70 mg kg^−1^, respectively) (Fig. 3c). The concentration of this metal in aboveground plant organs (stem and leaves) was 19–53 times lower for stems and 69–123 times for leaves compared to roots (Fig. 3a, b).

Thus, Cu accumulated mainly in the roots of the tested tree species. The highest mean content of Cu (59.7 mg per plant) in the whole biomass of the tested tree species was recorded in *A. platanoides*, where 58.2 mg per plant (approximately 98%) was accumulated in its roots. The abovementioned *Q. robur*, *T. cordata*, and *U. laevis* were also characterized by the high efficiency of Cu phytoextraction (26.1, 21.9, and 26.1 mg, respectively), with almost the same high accumulation of Cu in their roots (98, 95, and 98%, respectively). The content of Cu in the roots and stems of the control plants was much lower than in the treated seedlings. In the case of leaves, a significantly higher content of Cu was detected in the treated plants for *A. platanoides*, *Q. robur*, *T. cordata*, and *U. laevis*.

Lead was accumulated mainly in roots, as shown in Fig. 3d–f. Seedlings grown in mining sludge displayed significantly higher Pb concentrations in their roots and stems than in the same organs of the control plants. A significantly higher Pb content was observed in the leaves of treated *A. platanoides*, *B. pendula*, and *U. laevis* (3, 15, and 7 times, respectively) compared to the control. When comparing the total mean content of Pb in the whole biomass of the studied seedlings, the most effective accumulating tree species was *A. platanoides* (17.1 mg per plant) where Pb accumulation occurred mainly in its roots (16.4 g per plant—96% of total metal content). Promising results were also found for *Q. robur* (9.2 mg per plant) and *A. platanoides*, where the mean content of Pb accumulated in the roots was 96% (8.84 mg per plant) of the total biomass of Pb.

**Fig. 4:**
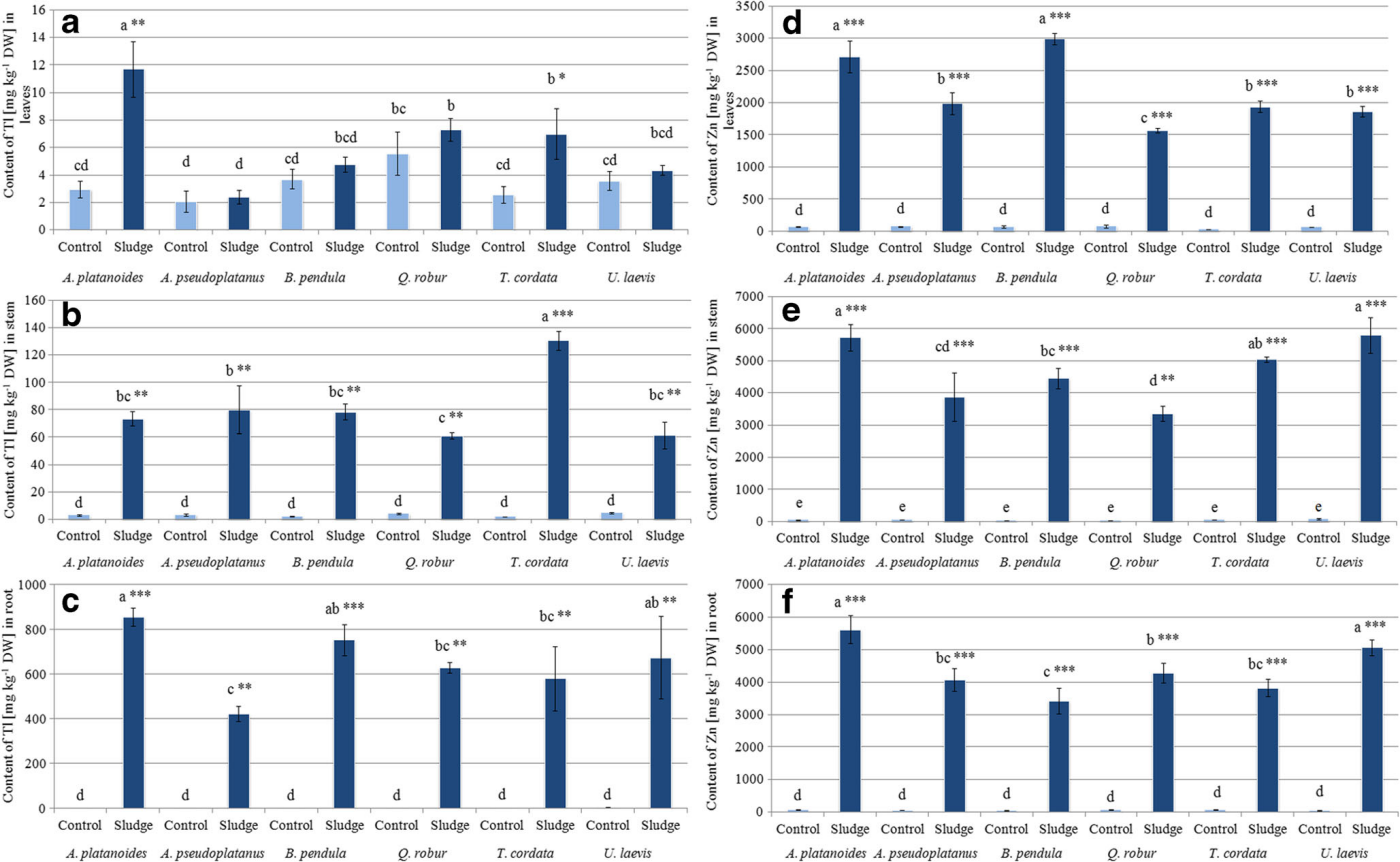
Content of thallium and zinc [mg kg^−1^ DW] in root, stem and leaves of tree species growing in soil (*Control*) and mining sludge (*Sludge*). Mean values (*n* = 3); *identical superscripts* (*a*, *b*, *c*…) denote no significant (*p* < 0.05) difference between mean content of Tl and Zn of all observations (separately for each organ of all control and treated tree species), according to Tukey’s HSD test (ANOVA); statistically significant differences between mean content of Tl and Zn of particular control and tree species treated with the *t* test are indicated by an *asterisk*

Planting the tested tree species on mining sludge led to significantly higher concentrations of Tl in the roots and stems of treated seedlings than in the control plants (Fig. 4b, c). A significantly higher content of this metal was also observed in the leaves of treated *A. platanoides* and *T. cordata* compared to the control (Fig. 4a). Considering that Tl accumulated mainly in roots (from 76% for *A. pseudoplatanus* to 93% for *A. platanoides* and *U. laevis*), the most effective phytoaccumulator of Tl was *A. platanoides*. The mean content of Tl in the whole biomass of this tree species was 27.7 mg per plant with up to 25.7 mg per plant in its roots. Three other tree species could also be promising for Tl phytoextraction: *B. pendula*, *Q. robur*, and *T. cordata*, with an average total content of Tl in the whole plant biomass of 11.0, 12.3, and 11.1 mg per plant, respectively. It is important to note that from these contents, 89, 92, and 83% of total Tl was accumulated in the roots.

Zinc was the only element whose content was significantly higher in all three organs of the tree seedlings growing in mining sludge than in the control plants (Fig. 4d–f). All the tested tree species showed high Zn phytoextraction ability. Unlike all the previously discussed elements, Zn was predominantly accumulated in shoots (stems and leaves) in all the tested tree species. *A. platanoides* showed the highest amount of Zn in its roots and stems (5626 ± 428 and 5732 ± 413 mg kg^−1^, respectively) and *U. laevis* 5068 ± 236 (roots) and 5801 ± 550 mg kg^−1^ (stems) (Fig. 4b). The content of Zn in the roots and stems of the other investigated tree species was lower but still very high (over 3200 mg kg^−1^). The highest content of Zn in leaves was observed in *A. platanoides* and *B. pendula* (2714 ± 251 and 2992 ± 88 mg kg^−1^, respectively). When comparing all six tree species as regards the total content of Zn in their whole biomass, the most effective accumulating plant was *A. platanoides* with a mean content of 333 mg per plant and with 164 mg of Zn per plant (almost 50% of total Zn) accumulated in the aboveground parts of this species. For three other tree species, *B. pendula*, *Q. robur*, and *T. cordata*, the average of total Zn content in the whole biomass was as follows: 117, 133, and 132 mg per plant, which corresponds to 72, 56, and 71% of total Zn content accumulated in shoots. These results indicate an effective translocation of Zn from the root system to the aboveground parts.

When comparing biomass and content of elements in the studied tree species, the most effective accumulator of all the six investigated metals/metalloid was *A. platanoides*, characterized by the highest mean content of As, Cd, Cu, Pb, Tl, and Zn.

### Phytoextraction efficiency

**Fig. 5:**
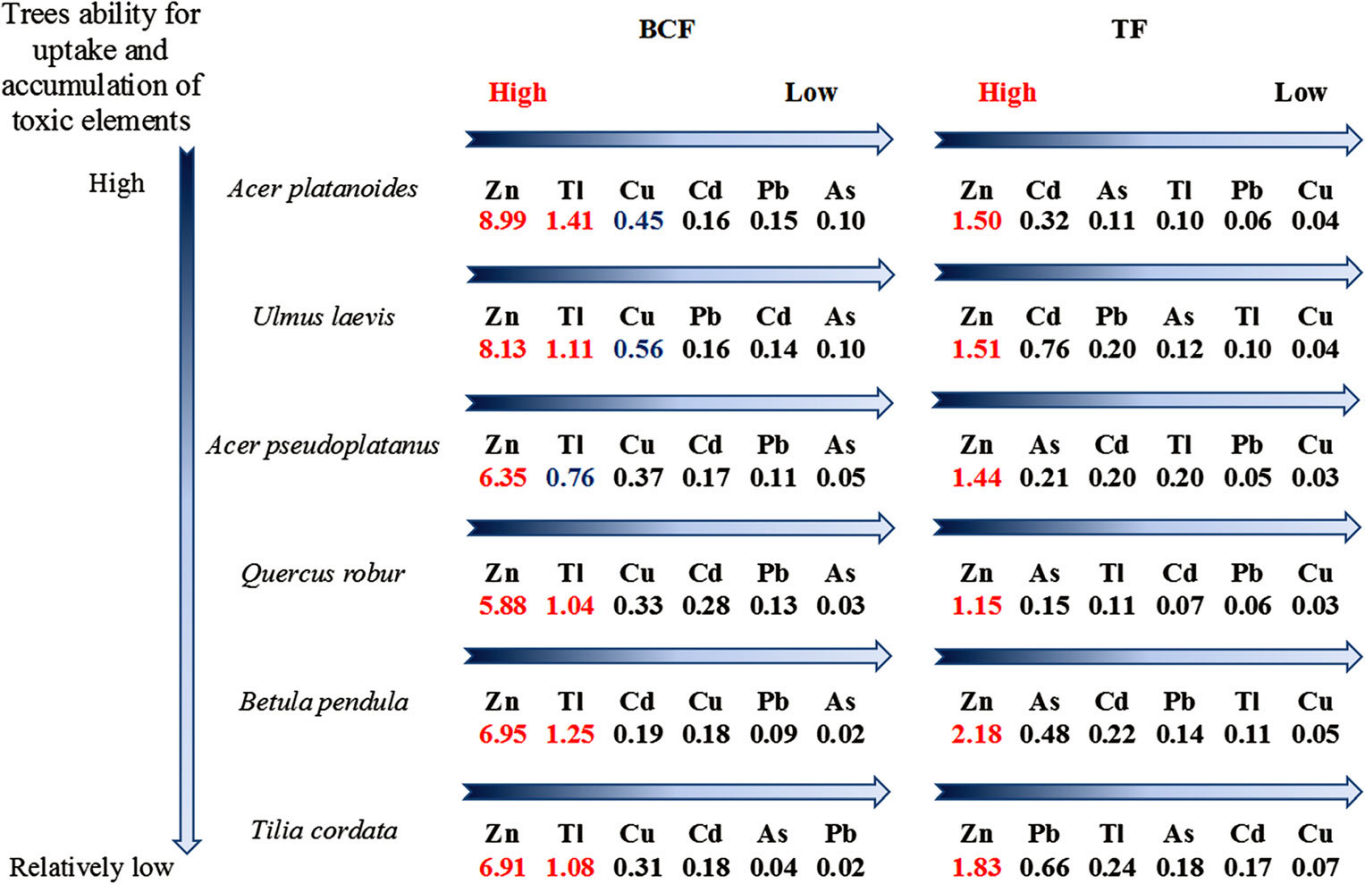
Bioaccumulation factor (BCF) and translocation factor (TF) values for studied tree species

All the tested tree species displayed the highest phytoextraction efficiency for Zn. The BCF for this element was always higher than 1 (Fig. 5). The highest BCF for Zn was found for *A. platanoides* (8.99), followed by *U. laevis* (8.13), *B. pendula* (6.95), *T. cordata* (6.91), *A. pseudoplatanus* (6.35), and *Q. robur* (5.88). *B. pendula*, where the TF was 2.18, was the most effective in Zn translocation from roots to shoots (Fig. 5). However, all the tested tree species showed a TF higher than 1 for Zn. Importantly, *A. platnoides*, which showed the highest BCF for Zn, also had a quite high TF = 1.5 for this element.

Promising phytoextraction data were also obtained for Tl. All the tested tree species had a BCF > 1 for this element, with the exception of *A. pseudoplatanus* with a BCF of 0.76. The best Tl accumulator was again *A. platanoides* (BCF = 1.41), followed by *B. pendula* (1.25), *U. laevis* (1.11), *T. cordata* (1.08), and *Q. robur* (1.04).

The BCF and TF for the other analyzed elements were below 1. The best accumulator for Cu and Pb was *U. laevis* (BCF = 0.56 and 0.16, respectively), for Cd *Q. robur* (BCF = 0.28), and for As *A. platanoides* and *U. laevis* (both BCF = 0.10). The best tree species in the translocation of Cd to shoots was *U. laevis* (TF = 0.76), *T. cordata* for Pb and Cu (TF = 0.66 and 0.07, respectively), and *B. pendula* for As (TF = 0.48). However, with the exception of Zn, all other elements (As, Cd, Cu, Pb, and Tl) were predominantly accumulated in the roots of the analyzed tree species.

## Discussion

Reduction of biomass was observed for *A. platanoides* and *T. cordata* growing on contaminated substrates, whereas differences between treated and control plants were not significant in the other tree species. Evangelou et al. (2013) made similar observations, recording a reduction of biomass in *B. pendula* but not in *Q. robur*. However, the concentrations of metals in the substrates used in the two studies were different. Lower biomass of seedlings grown on mining sludge or other contaminated substrates is a common physiological reaction to pollutants (Sheetal et al. 2016).

The great potential of trees (especially *A. platanoides* L.) for the efficient phytoextraction of potentially toxic elements is evident. It is worth underlining that before the experiment, seedlings were grown in an environment free from the direct influence of pollutants. Thus, it is possible that using the same tree species but collected from a contaminated area could give even more efficient phytoextraction results, as described by Kirkey et al. (2012). In fact, the growth of trees on substrates polluted with heavy metals is usually related with the resistance of plants to toxic elements. It is likely that plants already adapted to growing in polluted environments can develop a higher resistance to some polluting elements. By comparing the obtained results with literature data, similar data can be found regarding the relatively high potential of *B. pendula* for Zn phytoextraction, with particularly effective translocation of this metal to leaves (Dmuchowski et al. 2014; Pulford et al. 2001). However, the higher content of Pb in *B. pendula* stems rather than leaves described by Evangelou et al. (2013) or the efficiency in As and Cd phytoextraction in leaves observed by Alagić et al. (2013) were not observed in our studies. Data presented in the present manuscript support our earlier conclusion (Mleczek et al. 2016, 2017) that the ability of tree species to target metal phytoextraction depends not only on plant extraction ability but also on substrate characteristics.

In our earlier studies (Mleczek et al. 2016), the same tree species were grown on mining sludge with chemical characteristics similar to those of the sludge used in this study, except for a higher As concentration (27,487 mg kg^−1^) but in controlled conditions. It that study, a markedly lower amount of metals and metalloids were accumulated in plants compared to the amounts accumulated in the present experiments. For example, the amount of As determined in plants in the present experiments was many times higher (maximum concentration of As in *A. platanoides* roots 1616 ± 152 mg kg^−1^, but in *B. pendula* 188 ± 22 mg kg^−1^) than in previous ones (Mleczek et al. 2016), where the highest content (30.0 ± 1.3 mg kg^−1^) of this metalloid was observed in *B. pendula* roots. This value in *B. pendula* roots was over six times lower and clearly shows the significant influence of environmental factors such as rainfall and insulation to plant response (Magdziak et al. 2015; Alagić et al. 2013). These results suggest that plant responses in controlled conditions can be completely different to their responses in more natural environments (Stoltz and Greger 2002; Mleczek et al. 2009). In the current research, we observed significantly higher As phytoextraction, in spite of its lower concentration in mining sludge, in comparison to the previous paper. This result contrasts with most literature data (Makgalaka-Matlala et al. 2008; Gomes et al. 2012). It is likely that in the case of such high As concentrations in the substrate, combined with significant changes of macroelement content in plant organs (Supplementary data), both As phytoextraction and translocation efficiency can be modified. Another possibility may be the different ratio of As(III) to As(V) forms, which in our previous paper (Mleczek et al. 2016) were 2178 and 25,300 mg kg^−1^ DW, respectively. The greater concentration of As(V) in mining sludge could be the cause of the higher phytoextraction of As, especially in roots, as described in Makgalaka-Matlala et al. (2008) and Budzyńska et al. (2017).

All the tested tree species were able to uptake and translocate more than one metal from roots to shoots. However, the evaluation of the efficiency and selection of plants for phytoremediation purposes also depends on the extent of the BCF and TF. Only plants that show BCF and TF > 1 could be considered as good candidates for phytoextraction (Lorestani et al. 2011). BCF and TF > 1 are also characteristic for hyperaccumulators (Ali et al. 2013). Our results showed that all the tested tree species meet the requirements for Zn phytoextraction since their BCF and TF were higher than 1. The highest BCF for Zn was found for *A. platanoides* (8.99), while its TF was 1.5.

We can conclude that because of its vitality, tolerance, and the ability to uptake and translocate Zn to its aboveground parts, *A. platanoides* is a promising species for Zn phytoextraction from mining sludges. Similar abilities for Zn phytoextraction were present in *U. laevis* (BCF = 8.13 and TF = 1.51) followed by *A. pseudoplatanus* (BCF = 6.35, TF = 1.44). As a comparison, *A. pseudoplatanus* plants grown on a substrate with a similar content of Zn (1194 mg kg^−1^) accumulated a markedly lower amount of this metal, 109 mg kg^−1^ (Pourrut et al. 2011), while in our experiment, *A. pseudoplatanus* accumulated 2000 mg kg^−1^ and *A. platanoides* 2700 mg kg^−1^. Similarly to our results, *A. pseudoplatanus* showed the highest vitality compared to other plant species when growing on amended soil (Pourrut et al. 2011). In other experiments conducted on mine tailings containing twice the amount of Zn (2975 mg kg^−1^) than the substrate in our experiment, *Quercus phellow* contained about 600 mg Zn kg^−1^ (Shi et al. 2016) while in our experiments, *Q. robur* contained 1500 mg kg^−1^. This indicates that the phytoextraction potential of tree species not only depends on their extraction ability and element content in the substrate but can vary according, for example, to substrate physicochemical features and element availability.

An interesting aspect of the results included in this paper concerns the high efficiency of Tl phytoextraction. Tallium, usually present in low concentrations in soil, is also accumulated with low efficiency in plant organs (Madejón et al. 2007). Interestingly, almost all the tested tree species in our experiment also fulfilled the requirements for Tl phytoextraction. The BCF of all tree species for this element was higher than 1, and the best Tl accumulator was *A. platanoides* (BCF = 1.41). The content of Tl in whole-plant dry matter hyperaccumulators such as *Iberis intermedia* and *Biscutella laevigata* (Brassicaceae) described by Anderson et al. (1999) was 0.4 and 1.5%, respectively, values many times higher than those described in the present research. However, even if the plants used in our research cannot be considered hyperaccumulators, the trees grown on the highly polluted sludge were able to extract this metal effectively, although accumulation is mostly limited to roots, with TF values ranging from 0.1 (for *A. platanoides*) to 0.24 (for *T. cordata*).

For other elements, such as Cu, Cd, Pb, and As, the BCF and TF were lower than 1 in all the tested trees. However, a high level of metals and metalloids in the substrate could result in BCF < 1, even though the level of accumulated elements in the plant tissues is high. This occurred for example in ultramafic soils containing 3000 mg kg^−1^ Ni (Ali et al. 2013). In our experiments, we detected an extremely high concentration of As (over 18 g kg^−1^) in the mining sludge. This might explain the relatively low BCF of the analyzed tree species (e.g., 0.1 for *A. platanoides* and 0.02 for *T. cordata*). Nevertheless, the content of As in plant tissues was rather high, e.g., for *A. platanoides* it was 1770 mg kg^−1^.

The extremely low TF, e.g., in *A. platanoides* −0.04, together with a relatively high BCF (e.g., for *A. platanoides* 0.45) detected for Cu showed that this metal was taken up from the substrate but predominantly accumulated in the roots, similarly to what happens in other plants (Shi et al. 2016; Pignatelli et al. 2012). Generally, among the six analyzed metals and metalloids, Cu was the element least transported from roots to stems in all the investigated tree species. A similar tendency was noted for Pb, which is in accordance with other literature data (e.g., Rabeda et al. 2015; Tangahu et al. 2011; Madejón and Lepp 2007).

## Conclusions

Among the numerous factors that influence the phytoextraction efficiency of a particularly toxic element from polluted substrates (e.g., mining sludge, flotation tailings, or metalliferous soils), the selection of plants that can survive and effectively accumulate that element is essential. The results presented in this paper indicated the great potential of trees, especially *A. platanoides*, to carry out the effective phytoextraction of As, Cu, Pb, Tl, and Zn (52.4, 59.7, 17.1, 27.7, and 333 mg per plant, respectively) and to readily adapt to the presence of extremely high concentrations of metals (e.g., As, Cu, Pb, Tl, and Zn amounts of 18, 4.5, 3.9, 0.7, and 1.6 g kg^−1^, respectively) in a mining sludge substrate.

On the other hand, our results showed that the phytoremediative potential of the rest of the studied tree species is limited to the phytoextraction of selected elements only. For this reason, the proper selection of tree species is a factor that can strongly influence the efficiency of heavy metal phytoextraction. This is especially important when we decide to cultivate a great number of plants on a polluted area.

The described effective phytoextraction of toxic elements such as As (*U. laevis* 18.5 mg per whole plant) or Tl (*Q. robur* 12.3 mg per whole plant) gives indications about the possible use of the selected tree species for phytoremediative purposes. We expect that an experiment lasting longer than 90 days would give better phytoextraction results than those presented in this paper and would better point out the high potential of these long-lived plants to decontaminate polluted substrates.

## Electronic supplementary material

Fig S1 (GIF 46 kb).

High resolution image (TIFF 7294 kb).

Fig S2 (GIF 45 kb).

High resolution image (TIFF 6686 kb).

Fig S3 (GIF 71 kb).

High resolution image (TIFF 12306 kb).

ESM 1 (DOCX 85 kb).
