# Supplementary figures and images for: Phytoextraction of potentially toxic elements by six tree species growing on hazardous mining sludge
Source: Environ Sci Pollut Res Int. 2017 Aug 9;24(28):22183–95. doi: 10.1007/s11356-017-9842-3 (PMC5629231; doi:10.1007/s11356-017-9842-3)

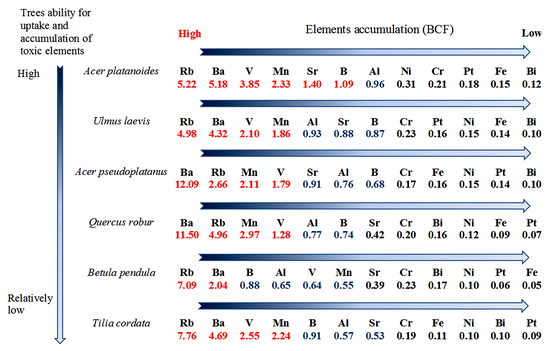

Supplement: Supplementary file 1 — (GIF 46 kb). [file 11356_2017_9842_Fig6_ESM.gif]

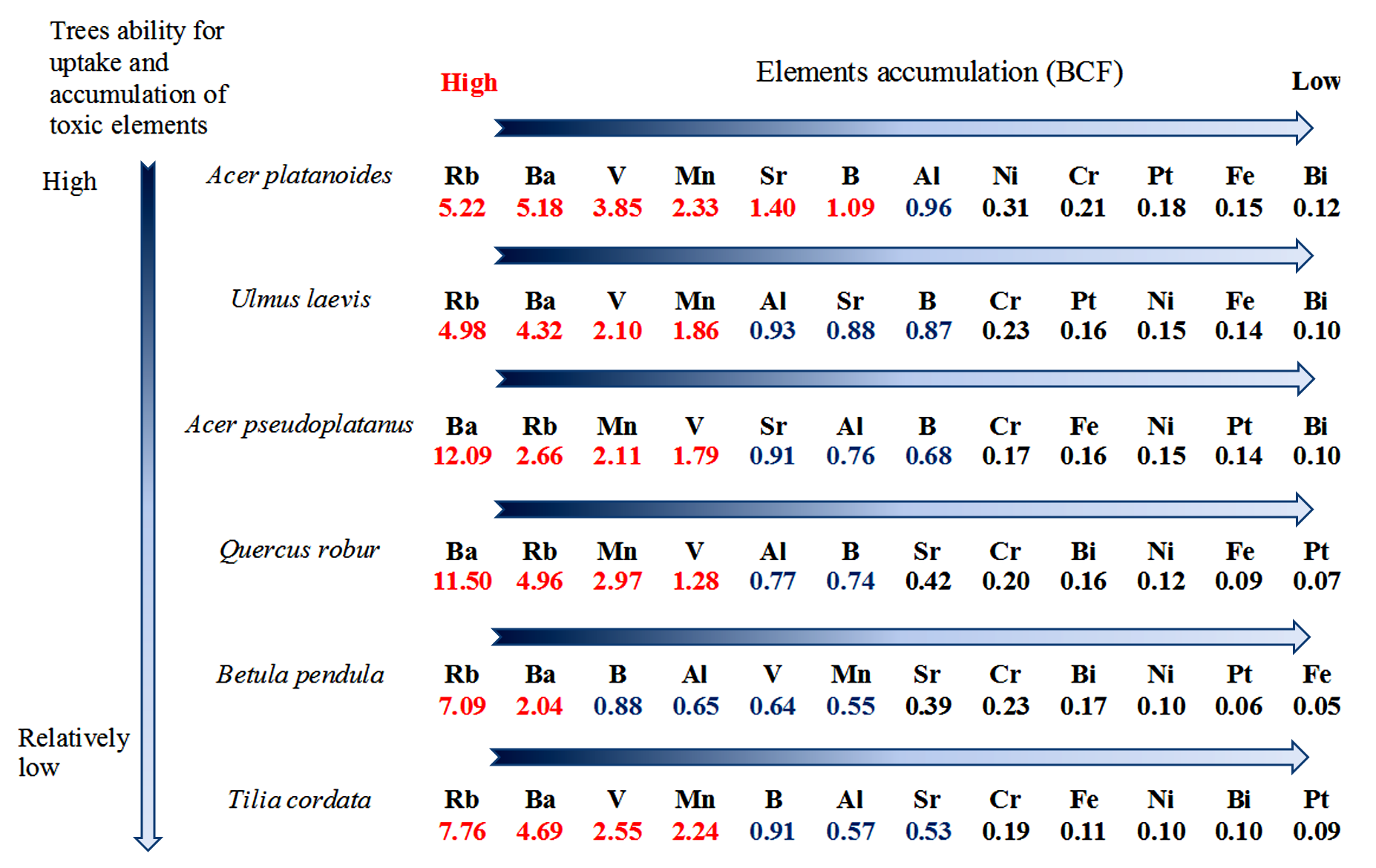

Supplement: Supplementary file 2 — High resolution image (TIFF 7294 kb). [file 11356_2017_9842_MOESM1_ESM.tif]

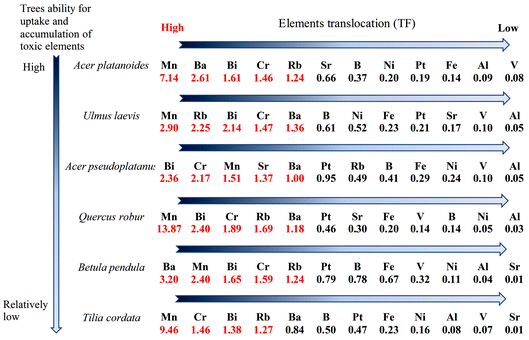

Supplement: Supplementary file 3 — (GIF 45 kb). [file 11356_2017_9842_Fig7_ESM.gif]

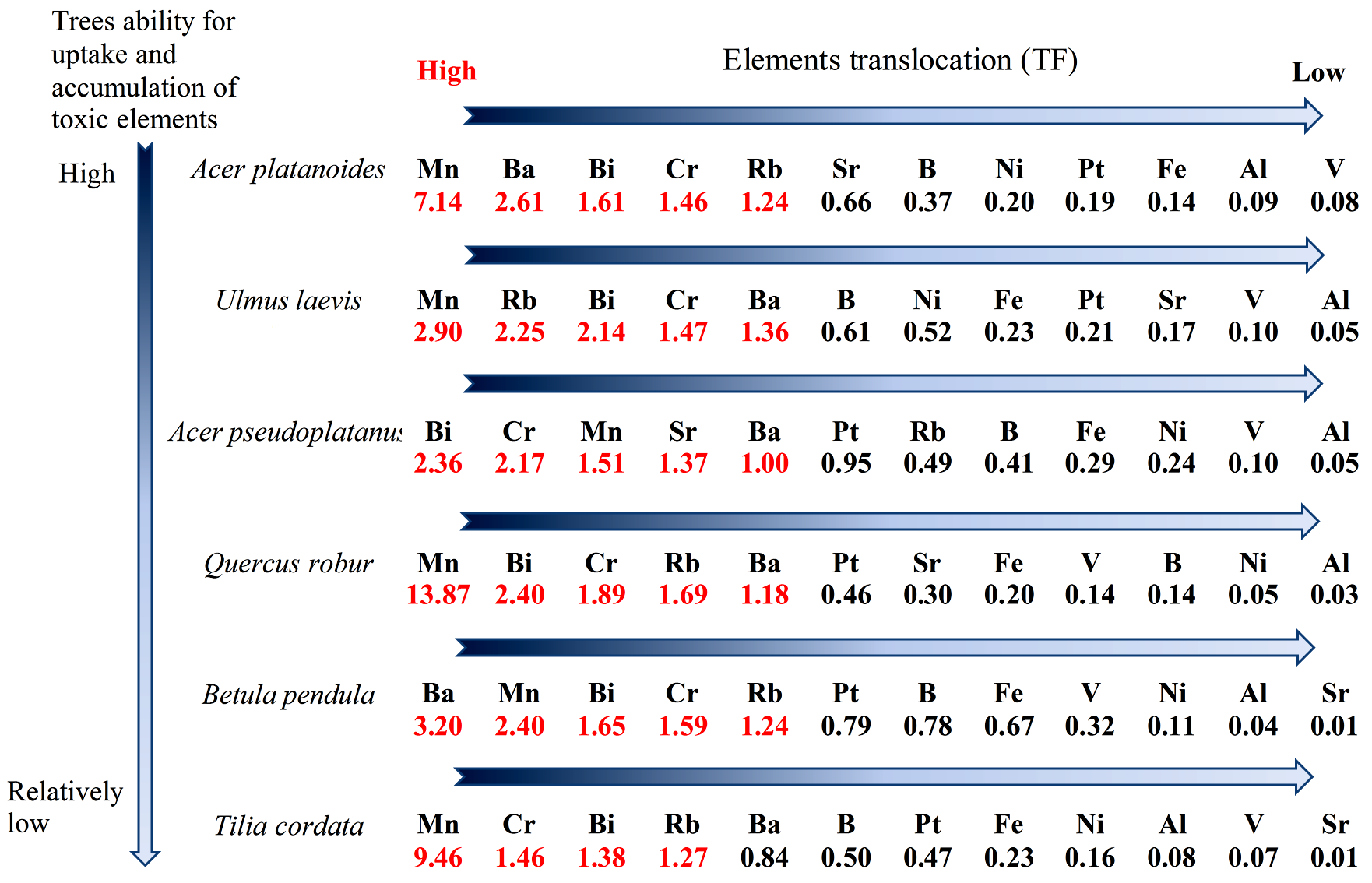

Supplement: Supplementary file 4 — High resolution image (TIFF 6686 kb). [file 11356_2017_9842_MOESM2_ESM.tif]

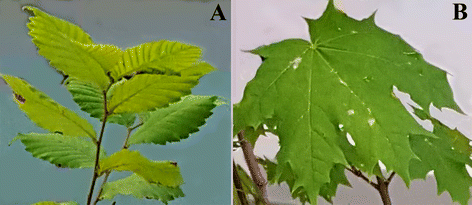

Supplement: Supplementary file 5 — (GIF 71 kb). [file 11356_2017_9842_Fig8_ESM.gif]

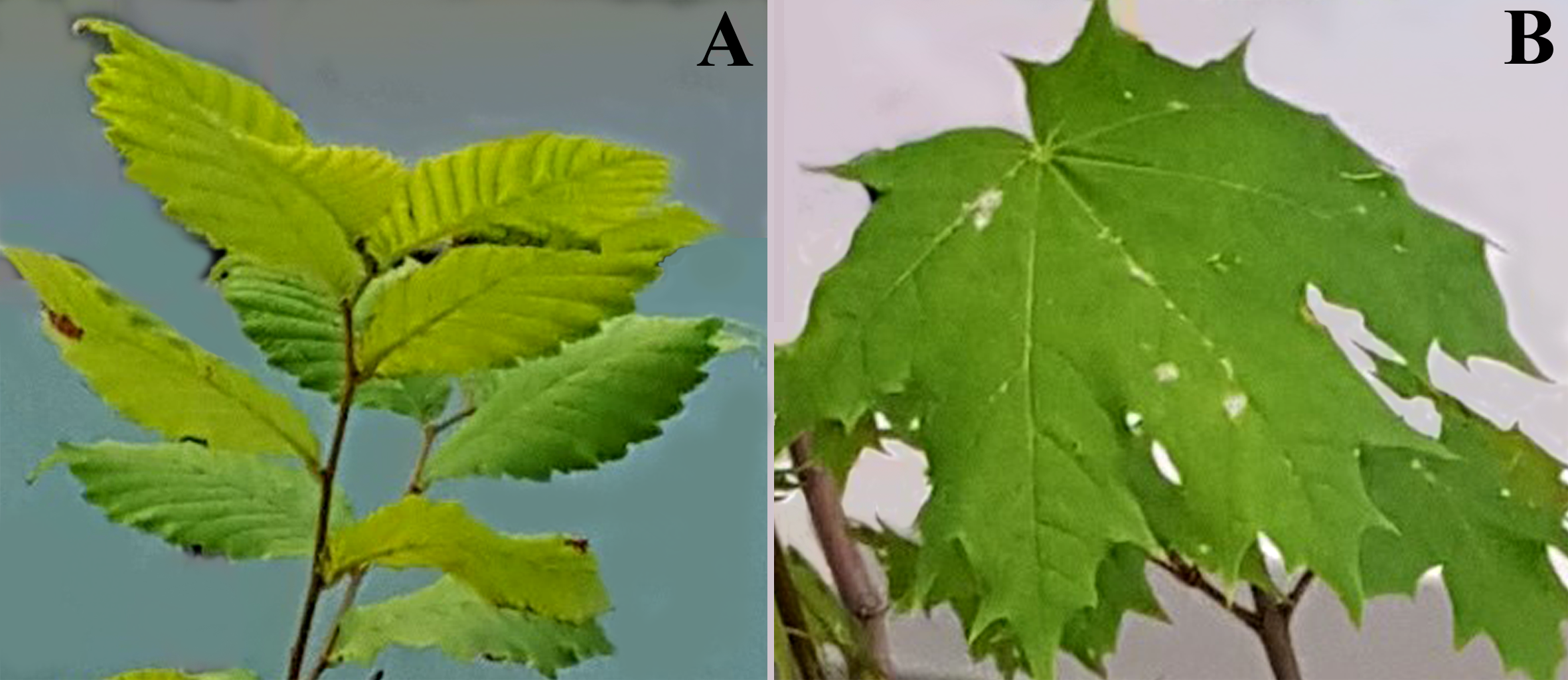

Supplement: Supplementary file 6 — High resolution image (TIFF 12306 kb). [file 11356_2017_9842_MOESM3_ESM.tif]
