# Supplementary material for: Phytoextraction of potentially toxic elements by six tree species growing on hazardous mining sludge
Source: Environ Sci Pollut Res Int. 2017 Aug 9;24(28):22183–95. doi: 10.1007/s11356-017-9842-3 (PMC5629231; doi:10.1007/s11356-017-9842-3)
Supplement: Supplementary file 7 — (DOCX 85 kb). [file 11356_2017_9842_MOESM4_ESM.docx]

Supplementary material for:

**"Phytoextraction of potentially toxic elements by six tree species growing on hazardous mining sludge"**

Mirosław Mleczek^1*^, Piotr Goliński^1^, Magdalena Krzesłowska^2^, Monika Gąsecka^1^, Zuzanna Magdziak^1^,

Paweł Rutkowski^3^, Sylwia Budzyńska^1^, Tomisław Kozubik^1,4^, Zbigniew Karolewski^5^, Przemysław Niedzielski^6^

^1^ *Poznań University of Life Sciences, Department of Chemistry, Wojska Polskiego 75,*

*60-625 Poznań,* *Poland*

^2^ *Adam Mickiewicz University in Poznań, Faculty of Biology, Laboratory of General Botany, Umultowska 89,*

*61-614* ***Poznań, Poland***

^3^ *Poznań University of Life Sciences, Department of Forest Sites and Ecology*,

*Wojska Polskiego 71F, 60-625 Poznań, Poland*

^4^ *Energetyka S.A. M. Skłodowskiej-Curie 58, 59-301 Lubin*

^5^ *Poznan University of Life Sciences, Department of Phytopathology, Dąbrowskiego 159, 60-594 Poznań, Poland*

^6^ *Adam Mickiewicz University in Poznań, Faculty of Chemistry, Umultowska 89B,*

*61-614* ***Poznań, Poland***

Supplementary Tables

**Table S1**. Content [mg kg^-1^ DW] of selected metals in root of tested tree species

**Table S2**. Content [mg kg^-1^ DW] of selected metals in stem of tested tree species

**Table S3**. Content [mg kg^-1^ DW] of selected metals in leaf of tested tree species

**Table S4.** Validation data for elemental analysis by ICP-OES

**Table S5.** Traceability studies for certified reference materials

**Table S1**. Content [mg kg^-1^ DW] of selected metals in root of tested tree species

| Tree species | | Al | B | Ba | Bi | Ca* | Cr | Fe | K* |
| --- | --- | --- | --- | --- | --- | --- | --- | --- | --- |
| *A. platanoides* | c | 154^c^±1 | 11^c^±1 | 5.8^de^±0.4 | 0.9^d^±0.1 | 2.3^f^±0.4 | 5.9^d^±0.9 | 739^ef^±106 | 2.9^cde^±0.1 |
|  | ms | 1772^a^±123 | 345^a^±12 | 17.2^cd^±2.7 | 13.7^ab^±2.0 | 11.7^a^±2.1 | 106.6^ab^±13.4 | 4087^a^±193 | 3.1^bcde^±0.2 |
| *A. pseudoplatanus* | c | 145^c^±27 | 10^c^±3 | 4.1^e^±0.9 | bDL | 4.5^def^±0.2 | 7.1^d^±1.8 | 217^f^±41 | 5.5^a^±0.1 |
|  | ms | 1445^ab^±67 | 210^b^±30 | 72.6^a^±10.9 | 8.6^c^±1.1 | 7.1^bc^±0.5 | 68.8^c^±7.3 | 3935^a^±249 | 1.4^ef^±0.1 |
| *B. pendula* | c | 257^c^±16 | 11^c^±1 | 7.7^de^±1.0 | 0.6^d^±0.2 | 3.5^ef^±0.4 | 3.7^d^±0.4 | 1214^de^±129 | 4.4^abc^±0.4 |
|  | ms | 1259^b^±87 | 212^b^±22 | 5.8^de^±1.0 | 18.2^a^±3.1 | 6.6^bcd^±0.5 | 113.2^ab^±9.7 | 894^ef^±58 | 2.8^cde^±0.1 |
| *Q. robur* | c | 311^c^±42 | 11^c^±2 | 5.7^de^±1.1 | 0.9^d^±0.1 | 3.1^ef^±0.1 | 6.7^d^±0.5 | 925^ef^±80 | 3.9^abc^±0.9 |
|  | ms | 1488^ab^±371 | 282^ab^±42 | 63.2^a^±3.7 | 13.6^ab^±2.6 | 5.6^cde^±0.5 | 86.3^bc^±3.2 | 2341^bc^±313 | 0.5^f^±0.1 |
| *T. cordata* | c | 241^c^±30 | 11^c^±2 | 4.3^e^±0.4 | 0.4^d^±0.1 | 4.1^def^±0.6 | 6.5^d^±1.0 | 1051^e^±262 | 3.3^bcde^±0.1 |
|  | ms | 1059^b^±310 | 262^ab^±33 | 30.6^b^±5.3 | 12.0^bc^±2.1 | 7.5^bc^±0.2 | 99.3^ab^±0.1 | 2838^b^±688 | 4.9^ab^±2.0 |
| *U. laevis* | c | 149^c^±4 | 10^c^±1 | 7.5^de^±1.2 | 0.3^d^±0.1 | 5.2^cde^±1.7 | 6.2^d^±1.3 | 1953^cd^±353 | 3.5^bcd^±0.3 |
|  | ms | 1760^a^±116 | 234^ab^±120 | 22.0^bc^±3.9 | 9.5^bc^±1.2 | 8.5^b^±0.3 | 118.3^a^±29.7 | 3665^a^±34 | 1.8^def^±0.2 |
| Tree species | | Mg* | Mn | Na | Ni | Pt | Rb | Sr | V |
| *A. platanoides* | c | 1.6^ef^±0.1 | 115^e^±9 | 845±57 | 4.6^c^±1.0 | 2.18^d^±1.04 | 3.48^d^±0.70 | 2.8^d^±0.2 | 6.5^e^±0.9 |
|  | ms | 6.3^a^±0.9 | 181^de^±31 | 1099±162 | 93.8^a^±15.6 | 56.19^a^±10.27 | 20.53^bc^±2.31 | 53.4^a^±9.7 | 111.3^a^±10.9 |
| *A. pseudoplatanus* | c | 0.6^f^±0.1 | 149^e^±18 | 175±19 | 1.3^c^±0.3 | 2.99^d^±0.76 | 2.70^d^±1.13 | 1.4^d^±0.4 | 3.4^e^±0.5 |
|  | ms | 4.3^b^±0.3 | 534^a^±49 | 949±128 | 43.1^b^±11.6 | 26.19^b^±3.59 | 15.80^c^±1.84 | 24.2^bc^±5.2 | 50.4^cd^±1.6 |
| *B. pendula* | c | 2.0^de^±0.1 | 134^e^±14 | 521±10 | 5.5^c^±0.8 | 2.26^d^±0.70 | 2.46^d^±0.37 | bDL | 1.8^e^±0.2 |
|  | ms | 3.8^bc^±0.9 | 102^e^±15 | 2511±230 | 31.6^b^±6.5 | 12.20^cd^±1.57 | 27.99^ab^±6.81 | 24.4^bc^±2.8 | 15.1^e^±2.9 |
| *Q. robur* | c | 2.6^cde^±0.2 | 284^cd^±81 | 298±43 | bDL | 1.67^d^±0.55 | 2.62^d^±0.97 | bDL | 3.5^e^±0.8 |
|  | ms | 4.0^b^±0.5 | 127^e^±8 | 547±53 | 41.2^b^±5.2 | 18.55^bc^±1.99 | 16.27^c^±3.58 | 20.7^c^±3.8 | 34.8^d^±6.5 |
| *T. cordata* | c | 1.7^def^±0.2 | 390^b^±53 | 396±19 | 2.1^c^±0.6 | 1.86^d^±0.42 | 2.12^d^±0.24 | bDL | 4.4^e^±0.7 |
|  | ms | 7.1^a^±0.1 | 136^e^±31 | 758±72 | 31.9^b^±6.8 | 23.82^bc^±5.26 | 30.15^a^±3.21 | 33.4^b^±5.4 | 74.0^b^±13.8 |
| *U. laevis* | c | 3.0^bcd^±0.5 | 340^bc^±15 | 1200±143 | 3.5^c^±0.6 | 1.31^d^±0.29 | 2.90^d^±0.20 | 1.5^d^±0.2 | 6.7^e^±1.2 |
|  | s | 6.0^a^±0.2 | 302^bc^±14 | 1145±7 | 35.2^b^±5.9 | 50.17^a^±8.09 | 13.52^c^±1.38 | 47.1^a^±5.6 | 59.1^bc^±8.7 |

Mean values (n=3) ± standard deviations; a, b, c… – different letters in column, differ significantly at P=0.01 (Tukey test); c – control (soil), ms – mining sludge; bDL – below detection limit; * - content in g kg^-1^.

**Table S2**. Content [mg kg^-1^ DW] of selected metals in stem of tested tree species

| Tree species | | Al | B | Ba | Bi | Ca | Cr | Fe | K |
| --- | --- | --- | --- | --- | --- | --- | --- | --- | --- |
| *A. platanoides* | c | 15^c^±4 | 5^d^±1 | 4.6^de^±0.5 | 1.3^e^±0.1 | 3.5^bcde^±0.4 | 7.0^c^±0.5 | 109^e^±21 | 1.9^bcd^±0.1 |
|  | ms | 97^a^±6 | 66^b^±12 | 24.8^b^±1.3 | 11.0^bc^±1.8 | 7.1^a^±0.6 | 81.6^b^±6.9 | 258^cde^±68 | 2.7^bc^±0.2 |
| *A. pseudoplatanus* | c | 48^bc^±10 | 3^d^±1 | 6.7^cde^±0.7 | bDL | 4.3^bc^±0.9 | 4.7^c^±1.0 | 164^de^±32 | 2.4^bcd^±0.4 |
|  | ms | 39^bc^±12 | 58^b^±10 | 31.5^ab^±7.5 | 12.2^bc^±3.1 | 4.6^bc^±1.1 | 85.6^b^±6.2 | 740^a^±93 | 6.3^a^±1.0 |
| *B. pendula* | c | 35^bc^±7 | 5^d^±1 | 4.2^e^±0.5 | 0.6^e^±0.1 | 1.7^e^±0.1 | 2.5^c^±0.3 | 167^de^±10 | 2.0^bcd^±0.3 |
|  | ms | 24^bc^±3 | 35^c^±5 | 6.1^cde^±2.3 | 17.2^a^±2.8 | 2.5^de^±0.2 | 90.3^ab^±7.3 | 372^bcd^±8 | 1.3^cd^±0.2 |
| *Q. robur* | c | 50^bc^±11 | 7^d^±2 | 8.0^cde^±1.8 | 0.6^e^±0.2 | 3.8^bcd^±0.3 | 7.6^c^±0.2 | 146^de^±23 | 1.1^d^±0.1 |
|  | ms | 44^bc^±9 | 28^c^±3 | 37.6^a^±3.6 | 13.6^ab^±0.3 | 4.4^bc^±0.5 | 86.9^b^±9.7 | 317^cde^±18 | 1.9^bcd^±0.2 |
| *T. cordata* | c | 69^ab^±27 | 7^d^±2 | 5.3^de^±0.3 | 0.7^e^±0.1 | 3.0^cde^±0.4 | 5.8^c^±0.4 | 203^cde^±50 | 1.9^bcd^±0.1 |
|  | ms | 55^abc^±1 | 84^a^±12 | 13.9^c^±1.8 | 5.9^d^±0.6 | 4.8^b^±0.9 | 87.1^b^±10.2 | 417^bc^±15 | 6.7^a^±0.3 |
| *U. laevis* | c | 56^abc^±39 | 5^d^±2 | 8.1^cde^±0.6 | 0.5^e^±0.1 | 4.3^bcd^±0.8 | 6.8^c^±0.9 | 220^cde^±81 | 2.0^bcd^±0.3 |
|  | s | 33^bc^±9 | 36^c^±1 | 12.3^cd^±0.6 | 8.3^cd^±0.4 | 5.2^b^±0.4 | 104.0^a^±2.0 | 572^b^±23 | 3.3^b^±0.6 |
| Tree species | | Mg | Mn | Na | Ni | Pt | Rb | Sr | V |
| *A. platanoides* | c | 1.2^bcd^±0.2 | 90^fg^±5 | 597^cde^±58 | 0.2^e^±0.1 | 1.07^e^±0.73 | 2.2^cd^±0.4 | bDL | 2.2^b^±0.2 |
|  | ms | 4.3^ab^±0.4 | 460^b^±39 | 1989^a^±361 | 15.1^a^±3.3 | 6.88^b^±0.55 | 15.8^b^±2.7 | 23.0^a^±3.1 | 4.1^a^±0.7 |
| *A. pseudoplatanus* | c | 0.3^d^±0.1 | 166^def^±62 | 40^f^±1 | 3.5^cd^±0.8 | 1.24^e^±0.30 | bDL | 0.8^d^±0.1 | 2.2^b^±0.2 |
|  | ms | 3.7^abc^±0.3 | 645^a^±38 | 1076^bc^±82 | 6.8^b^±1.1 | 19.29^a^±1.65 | 7.7^c^±1.8 | 16.3^b^±1.7 | 3.3^ab^±0.7 |
| *B. pendula* | c | 2.7^abcd^±0.5 | 22^g^±2 | 263^ef^±17 | 1.6^cde^±0.2 | 0.60^e^±0.78 | 1.7^d^±0.2 | bDL | 2.0^b^±0.1 |
|  | ms | 1.6^bcd^±0.1 | 18^g^±2 | 2360^a^±308 | 2.0^cde^±0.5 | 5.43^bcd^±0.54 | 19.7^ab^±1.4 | bDL | 2.8^ab^±0.8 |
| *Q. robur* | c | 2.4^abcd^±0.1 | 286^c^±33 | 200^ef^±35 | bDL | 1.12^e^±0.95 | 3.2^cd^±0.3 | bDL | 2.0^b^±0.1 |
|  | ms | 3.9^abc^±0.1 | 266^cd^±38 | 902^bc^±126 | 1.5^cde^±0.3 | 4.12^cde^±0.52 | 16.7^ab^±4.3 | 3.8^cd^±0.8 | 2.5^b^±0.1 |
| *T. cordata* | c | 0.9^cd^±0.2 | 198^cde^±25 | 318^def^±43 | 0.4^de^±0.1 | 1.99^e^±1.10 | 2.4^cd^±1.1 | bDL | 2.2^b^±0.1 |
|  | ms | 5.5^a^±1.2 | 147^ef^±11 | 1338^b^±289 | 2.8^cde^±0.2 | 5.47^bcd^±0.50 | 22.8^a^±4.7 | bDL | 3.2^ab^±0.3 |
| *U. laevis* | c | 2.0^bcd^±0.6 | 144^ef^±18 | 102^ef^±12 | 2.6^cde^±0.4 | 3.62^de^±0.14 | 4.5^cd^±1.2 | 1.0^d^±0.2 | 3.0^ab^±1.2 |
|  | s | 3.0^abcd^±0.1 | 482^b^±62 | 813^cd^±50 | 4.3^bc^±0.3 | 6.16^bc^±0.71 | 20.0^ab^±1.7 | 4.6^c^±1.2 | 3.0^ab^±0.1 |

Mean values (n=3) ± standard deviations; a, b, c… – different letters in column, differ significantly at P=0.01 (Tukey test); c – control (soil), ms – mining sludge; bDL – below detection limit; * - content in g kg^-1^.

**Table S3**. Content [mg kg^-1^ DW] of selected metals in leaf of tested tree species

| Tree species | | Al | B | Ba | Bi | Ca | Cr | Fe | K |
| --- | --- | --- | --- | --- | --- | --- | --- | --- | --- |
| *A. platanoides* | c | 22^d^±2 | 4^e^±1 | 4.3^e^±0.4 | 1.3^d^±0.1 | 6.4^b^±0.7 | 8.4^e^±1.1 | 230^b^±70 | 6.3^fg^±0.7 |
|  | ms | 60^ab^±6 | 61^c^±3 | 20.1^b^±2.4 | 11.1^bc^±1.5 | 6.3^b^±0.1 | 73.6^bc^±6.9 | 297^ab^±28 | 11.3^b^±0.6 |
| *A. pseudoplatanus* | c | 28^d^±3 | 3^e^±2 | 6.9d^e^±1.5 | bDL | 9.0^a^±0.3 | 5.3^e^±2.2 | 162^b^±25 | 6.9^ef^±0.1 |
|  | ms | 29^d^±3 | 28^d^±4 | 41.0^a^±3.8 | 8.0^c^±0.8 | 4.7^bcd^±0.4 | 63.4^cd^±5.9 | 393^a^±154 | 9.1^cd^±0.8 |
| *B. pendula* | c | 31^cd^±6 | 4^e^±1 | 6.8^de^±1.6 | 0.7^d^±0.2 | 6.7^b^±0.6 | 3.3^e^±0.4 | 209^b^±27 | 5.6^fg^±0.4 |
|  | ms | 29^d^±3 | 131^a^±8 | 12.5^cd^±1.5 | 12.8^b^±2.2 | 6.2^b^±0.2 | 89.2^a^±4.6 | 227^b^±16 | 8.1^de^±0.4 |
| *Q. robur* | c | 47^bc^±8 | 6^e^±1 | 4.2^e^±0.5 | 1.7^d^±0.1 | 4.8^bc^±0.2 | 6.1^e^±0.4 | 207^b^±47 | 5.1^gh^±0.1 |
|  | ms | 4^e^±1 | 12^de^±2 | 37.2^a^±3.0 | 19.0^a^±1.7 | 2.6^d^±0.1 | 76.6^b^±1.5 | 154^b^±17 | 6.8^ef^±0.4 |
| *T. cordata* | c | 37^cd^±12 | 7^e^±1 | 4.4^e^±0.5 | 0.7^d^±0.2 | 3.7^cd^±0.6 | 4.2^e^±0.4 | 218^b^±12 | 3.7^h^±0.3 |
|  | ms | 32^cd^±2 | 47^c^±4 | 11.8^cd^±2.1 | 10.6^bc^±3.8 | 4.7^bc^±0.1 | 57.8^d^±5.0 | 237^ab^±15 | 14.4^a^±0.4 |
| *U. laevis* | c | 68^a^±5 | 4^e^±1 | 5.9^e^±0.6 | 0.7^d^±0.1 | 9.8^a^±1.1 | 7.4^e^±0.8 | 283^ab^±37 | 8.0^de^±0.7 |
|  | s | 62^ab^±8 | 106^b^±16 | 17.5^bc^±2.2 | 12.0^bc^±1.7 | 9.0^a^±0.4 | 69.6^bc^±5.4 | 269^ab^±32 | 9.9^bc^±0.4 |
| Tree species | | Mg | Mn | Na | Ni | Pt | Rb | Sr | V |
| *A. platanoides* | c | 8.4^a^±0.6 | 78^f^±5 | 586^c^±87 | bDL | 0.80^c^±0.25 | 1.1^d^±0.1 | bDL | 2.1^b^±0.1 |
|  | ms | 7.1^a^±1.7 | 832^c^±66 | 841^b^±85 | 3.3^bc^±0.3 | 4.22^ab^±0.63 | 9.7^b^±0.8 | 12.2^b^±2.1 | 4.3^a^±0.8 |
| *A. pseudoplatanus* | c | 0.8^d^±0.1 | 109^f^±12 | 51^f^±16 | 1.2^cd^±0.7 | 0.36^d^±0.11 | bDL | bDL | 1.8^b^±0.2 |
|  | ms | 4.1^b^±0.4 | 160^ef^±29 | 49^f^±7 | 3.6^bc^±0.7 | 5.69^a^±0.76 | bDL | 16.8^a^±3.4 | 2.0^b^±0.2 |
| *B. pendula* | c | 8.1^a^±0.3 | 21^f^±6 | 313^d^±15 | 2.5^bcd^±1.4 | 1.47^c^±0.32 | 1.5^cd^±0.3 | bDL | 2.3^b^±0.2 |
|  | ms | 1.0^d^±0.1 | 225^ef^±18 | 134^f^±12 | 1.6^bcd^±0.1 | 4.18^ab^±0.33 | 14.9^a^±1.2 | bDL | 2.0^b^±0.1 |
| *Q. robur* | c | 7.8^a^±0.9 | 1431^a^±281 | 145^ef^±9 | bDL | 1.79^c^±0.58 | 2.5^cd^±0.6 | bDL | 2.3^b^±0.3 |
|  | ms | 4.3^b^±0.1 | 1490^a^±59 | 176^def^±6 | 0.5^d^±0.2 | 4.46^ab^±0.62 | 10.7^b^±1.4 | 2.4^cd^±0.6 | 2.5^b^±0.5 |
| *T. cordata* | c | 3.1^bc^±0.5 | 561^cd^±89 | 288^de^±62 | 0.5^d^±0.1 | 1.66^c^±1.17 | 2.3^cd^±0.3 | bDL | 2.2^b^±0.1 |
|  | ms | 3.4^b^±0.2 | 1137^b^±97 | 1320^a^±97 | 2.3^bcd^±0.3 | 5.62^a^±0.93 | 15.5^a^±2.4 | bDL | 2.1^b^±0.2 |
| *U. laevis* | c | 1.2^d^±0.3 | 20^f^±7 | 112^f^±24 | 4.1^b^±0.2 | 3.11^b^±0.34 | 3.7^c^±1.1 | 1.0^cd^±0.3 | 2.9^b^±0.7 |
|  | s | 1.3^cd^±0.1 | 395^de^±31 | 629^c^±51 | 13.9^a^±2.4 | 4.41^ab^±0.53 | 10.4^b^±1.7 | 3.6^c^±0.5 | 3.0^b^±0.6 |

Mean values (n=3) ± standard deviations; a, b, c… – different letters in column, differ significantly at P=0.01 (Tukey test); c – control (soil), ms – mining sludge; bDL – below detection limit; * - content in g kg^-1^.

**Table S4.** Validation data for elemental analysis by ICP-OES

|  | **wavelength** | **DL** | **range** | **uncertainty** |  | **wavelength** | **DL** | **range** | **uncertainty** |
| --- | --- | --- | --- | --- | --- | --- | --- | --- | --- |
|  | nm | mg kg^-1^ | mg kg^-1^ | **%** |  | nm | mg kg^-1^ | mg kg^-1^ | **%** |
| **Ag** | 328.068 | 0.0086 | DL-200 | 10.0 | **Na** | 588.995 | 0.26 | DL-1000 | 6.7 |
| **Al** | 394.401 | x | 200-4000 | 6.5 | **Na** | 589.592 | x | 1000-10000 | 3.4 |
| **Al** | 396.152 | 0.0053 | DL-200 | 2.9 | **Nb** | 313.078 | 0.0067 | DL-100 | 5.8 |
| **As** | 188.980 | 0.012 | DL-100 | 14.6 | **Nd** | 406.108 | 0.012 | DL-100 | 6.2 |
| **Au** | 197.742 | 0.0092 | DL-100 | 18.0 | **Ni** | 231.604 | 0.0092 | DL-200 | 8.0 |
| **B** | 249.772 | 0.047 | DL-200 | 3.4 | **Os** | 225.585 | 0.021 | DL-100 | 9.5 |
| **Ba** | 455.403 | 0.002 | DL-200 | 9.0 | **P** | 213.618 | 0.32 | DL-300 | 10.7 |
| **Be** | 313.042 | 0.026 | DL-100 | 12.0 | **P** | 253.561 | x | 300-4000 | 7.8 |
| **Bi** | 223.061 | 0.065 | DL-200 | 4.1 | **Pb** | 220.353 | 0.023 | DL-200 | 4.2 |
| **Ca** | 315.887 | x | 2000-10000 | 6.4 | **Pd** | 340.458 | 0.025 | DL-100 | 9.0 |
| **Ca** | 422.673 | 0.036 | DL-2000 | 2.8 | **Pr** | 417.939 | 0.032 | DL-100 | 9.8 |
| **Cd** | 214.439 | 0.0026 | DL-200 | 10.1 | **Pt** | 203.646 | 0.021 | DL-100 | 29.8 |
| **Ce** | 446.021 | 0.023 | DL-100 | 13.6 | **Rb** | 780.026 | 0.045 | DL-100 | 10.1 |
| **Co** | 238.892 | 0.0029 | DL-200 | 1.6 | **Re** | 197.248 | 0.032 | DL-100 | 5.0 |
| **Cr** | 267.716 | 0.0033 | DL-200 | 4.0 | **Rh** | 343.488 | 0.035 | DL-100 | 13.3 |
| **Cs** | 697.327 | 0.02 | DL-1000 | 12.1 | **Ru** | 240.272 | 0.021 | DL-100 | 9.4 |
| **Cu** | 327.395 | 0.0027 | DL-200 | 9.6 | **S** | 181.972 | 0.44 | DL-1000 | 15.7 |
| **Dy** | 364.540 | 0.023 | DL-100 | 6.7 | **Sb** | 206.834 | 0.012 | DL-100 | 14.0 |
| **Er** | 349.910 | 0.018 | DL-100 | 7.9 | **Sc** | 361.383 | 0.024 | DL-100 | 3.0 |
| **Eu** | 420.504 | 0.034 | DL-100 | 3.0 | **Se** | 196.026 | 0.011 | DL-100 | 16.4 |
| **Fe** | 238.204 | 0.0084 | DL-1000 | 1.6 | **Si** | 288.158 | 0.59 | DL-1000 | 19.2 |
| **Fe** | 261.382 | x | 100-4000 | 0.89 | **Sm** | 442.434 | 0.026 | DL-100 | 9.8 |
| **Ga** | 294.363 | 0.0097 | DL-200 | 3.4 | **Sn** | 283.998 | 0.067 | DL-100 | 12.3 |
| **Gd** | 342.246 | 0.034 | DL-100 | 6.1 | **Sr** | 460.733 | 0.0092 | DL-200 | 5.3 |
| **Ge** | 209.426 | 0.039 | DL-100 | 2.2 | **Ta** | 268.517 | 0.012 | DL-100 | 6.5 |
| **Hf** | 264.141 | 0.012 | DL-100 | 5.8 | **Tb** | 350.914 | 0.027 | DL-100 | 7.3 |
| **Hg** | 194.164 | 0.023 | DL-100 | 14.6 | **Te** | 214.282 | 0.011 | DL-100 | 5.5 |
| **Ho** | 348.484 | 0.031 | DL-100 | 11.9 | **Th** | 283.730 | 0.015 | DL-100 | 4.1 |
| **In** | 230.606 | 0.025 | DL-200 | 8.0 | **Ti** | 336.122 | 0.021 | DL-100 | 5.7 |
| **Ir** | 205.116 | 0.0097 | DL-100 | 6.5 | **Tl** | 190.794 | 0.024 | DL-200 | 7.3 |
| **K** | 766.491 | 0.34 | DL-1000 | 1.3 | **Tm** | 336.261 | 0.031 | DL-100 | 7.6 |
| **K** | 769.897 | x | 1000-10000 | 0.53 | **U** | 385.957 | 0.011 | DL-100 | 5.6 |
| **La** | 398.852 | 0.0086 | DL-100 | 6.8 | **V** | 292.401 | 0.018 | DL-100 | 6.2 |
| **Li** | 670.783 | 0.0044 | DL-200 | 16.6 | **W** | 207.912 | 0.027 | DL-1000 | 12.3 |
| **Lu** | 307.760 | 0.031 | DL-100 | 2.9 | **Y** | 361.104 | 0.031 | DL-100 | 6.2 |
| **Mg** | 279.553 | 0.008 | DL-500 | 9.7 | **Yb** | 328.937 | 0.014 | DL-100 | 3.8 |
| **Mg** | 285.213 | x | 500-5000 | 4.5 | **Zn** | 213.857 | 0.0022 | DL-200 | 3.5 |
| **Mn** | 257.610 | 0.0021 | DL-200 | 1.8 | **Zr** | 343.823 | 0.018 | DL-100 | 6.5 |
| **Mo** | 202.032 | 0.044 | DL-100 | 3.1 |  |  |  |  |  |

**Table S5.** Traceability studies for certified reference materials

|  | **Determined values** | | | | | | |  | **Certified values** | | | | | | |  | **Recovery** | | | | | | |
| --- | --- | --- | --- | --- | --- | --- | --- | --- | --- | --- | --- | --- | --- | --- | --- | --- | --- | --- | --- | --- | --- | --- | --- |
| mg/kg | CRM S-1 | CRM CS-M-1 | CRM CS-M-1 | CRM NCSDC | CRM San Jaoqain 2709 | CRM 405 | CRM 667 | mg/kg | CRM S-1 | CRM CS-M-1 | CRM CS-M-1 | CRM NCSDC | CRM San Jaoqain 2709 | CRM 405 | CRM 667 | % | CRM S-1 | CRM CS-M-1 | CRM CS-M-1 | CRM NCSDC | CRM San Jaoqain 2709 | CRM 405 | CRM 667 |
| **Ag** | <0.01 | <0.01 | <0.01 | <0.01 | 0.50 | 0.70 | 0.30 | **Ag** | x | x | x | 0.049 | x | x | x | **Ag** | x | x | x | 0 | x | x | x |
| **Al** | 7071 | 70 | 69 | 730 | 19930 | 21008 | 17191 | **Al** | x | x | x | 2000 | 73700 | 77900 | x | **Al** | x | x | x | 37 | 27 | 27 | x |
| **As** | 3.0 | 0.70 | 0.40 | 0.90 | 13 | 17 | 12 | **As** | 3.4 | 0.344 | 0.344 | 1.25 | 10.5 | 23.6 | x | **As** | 88 | 203 | 116 | 72 | 121 | 71 | x |
| **Au** | 0.80 | 0.90 | 0.80 | 0.90 | 1.2 | 1.5 | 1.3 | **Au** | x | x | x | x | x | x | x | **Au** | x | x | x | x | x | x | x |
| **B** | 8.6 | 2.1 | 2.0 | 34 | 48 | 42 | 47 | **B** | x | x | x | 38 | 74 | x | x | **B** | x | x | x | 90 | 64 | x | x |
| **Ba** | 38 | 1.8 | 1.8 | 13 | 384 | 51 | 75 | **Ba** | 304 | x | x | 18 | x | x | x | **Ba** | 13 | x | x | 74 | x | x | x |
| **Bi** | <0.01 | <0.01 | 0.10 | <0.01 | <0.01 | 0.35 | 0.65 | **Bi** | x | x | x | 0.023 | x | x | x | **Bi** | x | x | x | 0 | x | x | x |
| **Ca** | 1285 | 315 | 313 | 15208 | 12211 | 19074 | 19850 | **Ca** | 2600 | x | x | 16800 | 19100 | x | x | **Ca** | 49 | x | x | 91 | 64 | x | x |
| **Cd** | 0.40 | 0.20 | 0.20 | 0.70 | 0.80 | 1.2 | 1.1 | **Cd** | 0.3 | 0.273 | 0.273 | 0.38 | 0.371 | 0.73 | 0.67 | **Cd** | 133 | 73 | 73 | 184 | 216 | 158 | 157 |
| **Ce** | 28 | 0.30 | 0.20 | 5.9 | 31 | 43 | 21 | **Ce** | 44 | x | x | 2.2 | 42 | x | 56.7 | **Ce** | 63 | x | x | 268 | 74 | x | 36 |
| **Co** | 1.4 | 0.10 | 0.20 | 0.20 | 2.3 | 1.8 | 6.3 | **Co** | 3.9 | x | x | 0.41 | 12.8 | 13.7 | 23 | **Co** | 36 | x | x | 49 | 18 | 13 | 27 |
| **Cr** | 10 | 0.50 | 0.50 | 1.2 | 57 | 34 | 83 | **Cr** | 38 | x | x | 2.6 | 130 | 84 | 178 | **Cr** | 27 | x | x | 46 | 44 | 40 | 47 |
| **Cu** | 5.4 | 7.2 | 7.2 | 5.2 | 27 | 40 | 52 | **Cu** | 6.3 | x | x | 6.6 | x | 47.7 | 60 | **Cu** | 86 | x | x | 79 | x | 83 | 87 |
| **Dy** | 2.7 | <0.01 | <0.01 | <0.01 | 4.4 | 3.7 | 2.2 | **Dy** | x | x | x | 0.13 | 3 | x | 4.01 | **Dy** | x | x | x | 0 | 145 | x | 54 |
| **Er** | 126 | 1.3 | 1.2 | 33 | 618 | 713 | 247 | **Er** | x | x | x | x | x | x | 2.35 | **Er** | x | x | x | x | x | x | 10511 |
| **Eu** | 0.40 | <0.01 | <0.01 | <0.01 | 0.60 | 0.70 | 0.55 | **Eu** | 0.6 | x | x | 0.039 | 0.83 | 1.25 | 1 | **Eu** | 67 | x | x | 0 | 72 | 56 | 55 |
| **Fe** | 7853 | 99 | 97 | 826 | 19459 | 19655 | 20980 | **Fe** | 9880 | x | x | 1070 | 33600 | 37400 | 44800 | **Fe** | 79 | x | x | 77 | 58 | 53 | 47 |
| **Ga** | 2.0 | <0.01 | 0.10 | <0.01 | 8.2 | 8.9 | 7.2 | **Ga** | x | x | x | x | x | x | x | **Ga** | x | x | x | x | x | x | x |
| **Gd** | 2.9 | <0.01 | <0.01 | 0.30 | 6.4 | 7.4 | 7.7 | **Gd** | x | x | x | 0.19 | 3 | x | 4.41 | **Gd** | x | x | x | 158 | 212 | x | 173 |
| **Ge** | <0.01 | <0.01 | <0.01 | <0.01 | <0.01 | 0.30 | 0.05 | **Ge** | x | x | x | x | x | x | x | **Ge** | x | x | x | x | x | x | x |
| **Ho** | 0.10 | <0.01 | <0.01 | <0.01 | 0.05 | 0.05 | 0.10 | **Ho** | x | x | x | 0.033 | x | x | 0.8 | **Ho** | x | x | x | 0 | x | x | 13 |
| **In** | 0.20 | 0.80 | 0.70 | <0.01 | <0.01 | <0.01 | <0.01 | **In** | x | x | x | x | x | x | x | **In** | x | x | x | x | x | x | x |
| **Ir** | 3.0 | 0.60 | 1.1 | 1.3 | 14 | 13 | 14 | **Ir** | x | x | x | x | x | x | x | **Ir** | x | x | x | x | x | x | x |
| **K** | 1199 | 14562 | 14540 | 8613 | 3724 | 5287 | 4359 | **K** | 12500 | x | x | 9200 | 21100 | 24900 | x | **K** | 10 | x | x | 94 | 18 | 21 | x |
| **La** | 12 | 0.10 | 0.10 | 0.90 | 13 | 18 | 8.9 | **La** | 21 | x | x | 1.25 | 21.7 | 40.4 | 27.8 | **La** | 57 | x | x | 72 | 60 | 45 | 32 |
| **Li** | 6.2 | 0.10 | 0.10 | 3.0 | 44 | 65 | 44 | **Li** | x | x | x | 2.6 | x | 72 | x | **Li** | x | x | x | 115 | x | 90 | x |
| **Lu** | 0.20 | <0.01 | <0.01 | <0.01 | 0.65 | 0.70 | 0.80 | **Lu** | 0.3 | x | x | 0.011 | 0.3 | 0.468 | 0.325 | **Lu** | 67 | x | x | 0 | 217 | 150 | 246 |
| **Mg** | 1054 | 658 | 660 | 4158 | 4398 | 4043 | 4114 | **Mg** | 1550 | x | x | 4800 | 14600 | 12300 | x | **Mg** | 68 | x | x | 87 | 30 | 33 | x |
| **Mn** | 196 | 11 | 11 | 58 | 421 | 373 | 723 | **Mn** | 266 | x | x | 61 | 529 | 495 | 920 | **Mn** | 74 | x | x | 95 | 80 | 75 | 79 |
| **Mo** | 708 | 1327 | 982 | 1584 | 7227 | 2801 | 4097 | **Mo** | x | x | x | 0.28 | x | x | x | **Mo** | x | x | x | 565750 | x | x | x |
| **Na** | 122 | 223 | 222 | 7453 | 588 | 4592 | 5108 | **Na** | 4440 | x | x | 19600 | 12200 | x | x | **Na** | 3 | x | x | 38 | 5 | x | x |
| **Nd** | 14 | 0.50 | 0.60 | 55 | 40 | 68 | 82 | **Nd** | x | x | x | 1 | 17 | x | 25 | **Nd** | x | x | x | 5540 | 235 | x | 327 |
| **Ni** | 8.0 | 0.30 | 0.30 | 1.1 | 64 | 22 | 90 | **Ni** | 13 | x | x | 1.7 | 85 | 32.5 | 128 | **Ni** | 62 | x | x | 65 | 76 | 68 | 70 |
| **Pb** | 10.5 | 0.60 | 0.70 | 44 | 11 | 54 | 24 | **Pb** | 15 | 0.476 | 0.476 | 47 | 17.3 | 74.8 | 31.9 | **Pb** | 70 | 126 | 147 | 94 | 64 | 72 | 76 |
| **Pd** | <0.01 | <0.01 | <0.01 | <0.01 | <0.01 | <0.01 | <0.01 | **Pd** | x | x | x | x | x | x | x | **Pd** | x | x | x | x | x | x | x |
| **Pr** | 2.5 | 0.10 | 0.10 | <0.01 | 1.7 | 3.1 | 1.4 | **Pr** | x | x | x | 0.24 | x | x | 6.1 | **Pr** | x | x | x | 0 | x | x | 23 |
| **Pt** | 79 | 1.9 | 1.5 | 9.5 | 276 | 281 | 326 | **Pt** | x | x | x | x | x | x | x | **Pt** | x | x | x | x | x | x | x |
| **Re** | 0.20 | 0.10 | 0.20 | 0.20 | 0.10 | 0.10 | 0.20 | **Re** | x | x | x | x | x | x | x | **Re** | x | x | x | x | x | x | x |
| **Rh** | <0.01 | <0.01 | <0.01 | <0.01 | <0.01 | <0.01 | <0.01 | **Rh** | x | x | x | x | x | x | x | **Rh** | x | x | x | x | x | x | x |
| **Ru** | 1.2 | <0.01 | <0.01 | 0.10 | 5.5 | 6.6 | 5.2 | **Ru** | x | x | x | x | x | x | x | **Ru** | x | x | x | x | x | x | x |
| **Sb** | 0.40 | 0.20 | <0.01 | 0.30 | 4.2 | 1.5 | 2.5 | **Sb** | 0.5 | x | x | 0.095 | 1.55 | 1.81 | 0.96 | **Sb** | 80 | x | x | 316 | 271 | 80 | 260 |
| **Sc** | 2.0 | <0.01 | <0.01 | 0.20 | 6.0 | 3.9 | 4.7 | **Sc** | 4 | x | x | x | 11.1 | 13.52 | 13.7 | **Sc** | 50 | x | x | x | 54 | 29 | 34 |
| **Se** | <0.01 | 1.5 | 1.7 | 0.30 | 0.70 | 0.10 | 0.35 | **Se** | x | 1.37 | 1.37 | x | 1.5 | 0.44 | 1.59 | **Se** | x | 109 | 124 | x | 47 | 23 | 22 |
| **Sm** | <0.01 | 0.10 | <0.01 | <0.01 | <0.01 | 1.3 | 0.30 | **Sm** | 3.6 | x | x | 0.19 | 4 | 5.86 | 4.66 | **Sm** | 0 | x | x | 0 | 0 | 22 | 6 |
| **Sr** | 10 | 1.0 | 1.0 | 262 | 110 | 104 | 208 | **Sr** | 55 | x | x | 246 | x | 118 | x | **Sr** | 19 | x | x | 106 | x | 88 | x |
| **Te** | 0.60 | <0.01 | 0.70 | 0.40 | 2.6 | 3.1 | 3.3 | **Te** | x | x | x | x | 0.5 | x | x | **Te** | x | x | x | x | 520 | x | x |
| **Tl** | 0.20 | <0.01 | 0.10 | <0.01 | 0.80 | 0.50 | 0.65 | **Tl** | x | x | x | x | 0.58 | x | x | **Tl** | x | x | x | x | 138 | x | x |
| **Tm** | 0.90 | <0.01 | <0.01 | 0.10 | 3.7 | 3.9 | 4.5 | **Tm** | x | x | x | x | x | x | 0.326 | **Tm** | x | x | x | x | x | x | 1380 |
| **Y** | 5.6 | <0.01 | <0.01 | 0.40 | 8.8 | 8.8 | 9.7 | **Y** | x | x | x | 0.68 | 2 | x | x | **Y** | x | x | x | 59 | 438 | x | x |
| **Yb** | 0.60 | <0.01 | <0.01 | <0.01 | 1.2 | 1.1 | 1.2 | **Yb** | 2.5 | x | x | 0.063 | x | 3.04 | 2.2 | **Yb** | 24 | x | x | 0 | x | 35 | 52 |
| **Zn** | 37 | 60 | 60 | 52 | 84 | 224 | 132 | **Zn** | 35 | 60.94 | 60.94 | 55 | 103 | 279 | 175 | **Zn** | 105 | 99 | 99 | 95 | 81 | 80 | 76 |
